# Piezo‐Phototronic PVDF/HfO_2_/Nano‐Cu Heterostructured Thin Film for Flexible Self‐Powered Multimodal Sensing

**DOI:** 10.1002/advs.202518913

**Published:** 2025-12-07

**Authors:** Jiawei Gu, Qiongle Peng, Xuanqi Zhong, Yi Zheng, Zhiqiang Ma, Xiaoxian Song, Ruihuan Zhang, Bao Liu, Yanhu Zhang, Zhengbao Yang

**Affiliations:** ^1^ School of Mechanical Engineering Jiangsu University No. 301 Xuefu Road Zhenjiang 212013 China; ^2^ Department of Blood Transfusion Affiliated Hospital of Jiangsu University No. 438 Jiefang Road Zhenjiang 212000 China; ^3^ Department of Mechanical and Aerospace Engineering Hong Kong University of Science and Technology Hong Kong SAR 999077 China; ^4^ School of Mechanical Engineering and Automation Beihang University No. 37 Xueyuan Road Beijing 100191 China; ^5^ Automotive Engineering Research Institute Jiangsu University No. 301 Xuefu Road Zhenjiang 212013 China

**Keywords:** energy harvesting, heterostructure, multimodal sensing, piezoelectric, thin film

## Abstract

The pursuit of multifunctional sensors has intensified due to their potential for integrated, efficient operation. However, realizing a flexible material that combines simplicity in the fabrication process with high performance and multifunctionality remains a formidable challenge. In this study, a multi‐interface heterostructured nanofiber membrane composed of Polyvinylidene fluoride (PVDF), HfO_2_, and nano‐copper is fabricated, leveraging the synergistic interplay of piezoelectric and piezo‐phototronic effects for flexible, self‐powered sensing. The incorporation of hafnium oxide (HfO_2_) and nano‐Cu enhances the crystallinity of the piezoelectric *β*‐phase and improves the dielectric properties, substantially boosting electromechanical performance. The resultant piezoelectric sensor achieves an output voltage of 15V and maintains stability over 5000 cycles at 5 Hz, enabling precise detection of human motions. Meanwhile, the PVDF/HfO_2_‐based photodetector exhibits broadband sensitivity (from near‐UV to near‐IR), high photoresponsivity (5.67 mA W^−1^), high detectivity (4.14×10^11^ Jones), and rapid response (600 µs rising, 53 µs falling). Critically, the device demonstrates a 28.6% current enhancement under simultaneous light and pressure, highlighting a strong piezo‐phototronic effect. This work presents a multifunctional fiber film for dual‐mode sensing (pressure & light), with promising applications in wearable electronics and aerospace systems. Its straightforward fabrication and high performance offer a viable pathway toward next‐generation, flexible, self‐powered sensors.

## Graphical abstract

Electrospinning a composite of PVDF, HfO_2_, and nano‐Cu transforms the discrete precursors into a continuous nanofiber mesh, forming a multi‐interface heterostructure with embedded nanoparticles. The presence of HfO_2_ and nano‐Cu at the fiber interfaces induces a highly aligned β‐phase, which enhances piezoelectric crystallinity and improves the material's dielectric properties.

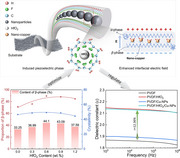

## 1 Introduction

Flexible multifunctional sensors, capable of simultaneously detecting multiple physical or environmental signals, have emerged as pivotal components for real‐time monitoring, healthcare diagnostics, industrial automation, and innovative electronics.^[^ ^1^, ^2^, ^3^, ^4^, ^5^, ^6^ ^]^ Current multifunctional sensor designs typically integrate discrete sensing units into compact assemblies; however, this approach imposes structural complexity, limited scalability, and compromised flexibility—particularly as functionality increases.^[^ ^7^, ^8^, ^9^ ^]^ Moreover, stringent fabrication requirements and elevated production costs often degrade device reliability and signal sensitivity, hindering widespread adoption.^[^ ^10^, ^11^, ^12^, ^13^ ^]^ Addressing these challenges necessitates the development of unified, flexible sensing materials that combine simplicity of fabrication with robust multifunctionality.^[^ ^14^, ^15^, ^16^ ^]^

Among emerging multifunctional sensors, these merging piezoelectric and optoelectronic functionalities hold particular promise due to their energy‐autonomous operation and broad sensing applicability.^[^ ^17^, ^18^ ^]^ For instance, PVDF/CeO_2_@PDA composite films enable dual‐mode force/light detection.^[^ ^19^ ^]^ The piezo‐phototronic effect boosts photodetector efficiency—wherein piezoelectric polarization modulates interfacial band structures to enhance charge separation—further improving photodetection efficiency.^[^ ^20^, ^21^, ^22^, ^23^ ^]^ Lai et al. demonstrated this effect in MAPbI_3_‐based photodetectors, achieving simultaneous gains in photocurrent magnitude and response speed.^[^ ^24^ ^]^ Such advances underscore the potential of self‐powered, multifunctional optoelectronic sensors leveraging synergistic material properties.

PVDF and its copolymers are widely employed in flexible piezoelectrics due to their inherent flexibility, chemical stability, and ease of processing.^[^ ^25^, ^26^, ^27^, ^28^, ^29^ ^]^ PVDF's activity primarily arises from its *β*‐phase (all‐trans TTTT conformation), distinguished by strong ferroelectric and pyroelectric responses.^[^ ^30^, ^31^, ^32^, ^33^, ^34^ ^]^ Conventional methods to enhance *β*‐phase content — such as mechanical stretching, thermal annealing, and high‐voltage poling are effective.^[^ ^35^, ^36^, ^37^, ^38^ ^]^ However, these methods introduce fabrication complexity and risks (e.g., dielectric breakdown).^[^ ^39^, ^40^, ^41^ ^]^ In contrast, electrospinning simultaneously aligns polymer dipoles and induces mechanical strain, obviating the need for post‐treatment.^[^ ^42^, ^43^, ^44^ ^]^ Complementary strategies include blending PVDF with nanofillers (e.g., piezoelectric ceramics, metal oxides, or conductive particles) to nucleate *β*‐phase crystallization^[^ ^45^, ^46^, ^47^, ^48^, ^49^, ^50^ ^]^ and enhance dielectric properties.^[^ ^51^, ^52^ ^]^ For example, nano‐copper offers high conductivity and cost‐effectiveness relative to noble metals, while HfO_2_ combines a high dielectric constant with ferroelectric switching and wide‐bandgap semiconductivity.^[^ ^53^, ^54^, ^55^ ^]^ These attributes make HfO_2_‐PVDF composites ideal for dual‐mode piezoelectric‐photoelectric sensing.

Here, we report a self‐powered, flexible, multifunctional sensor integrating HfO_2_ and nano‐copper into a PVDF nanofiber matrix via scalable electrospinning. The fillers synergistically enhance *β*‐phase crystallinity (nano‐copper through local electric field intensification; HfO_2_ via nucleation), while HfO_2_ enables piezoelectric‐photoelectric coupling. The resulting composite exhibits a 3.95‐fold increase in piezoelectric output (15 V) compared to pure PVDF, alongside broadband photoresponse (UV–IR) and a 28.6% current gain under concurrent pressure/illumination, confirming the piezo‐phototronic effect. Potential applications span wearable health monitors (e.g., tracking biomechanical motion and ambient light) and aerospace systems (e.g., real‐time structural health assessment of rovers). By unifying high performance, ease of fabrication, and multifunctionality, this work establishes a versatile platform for next‐generation sensors.

## 2 Results and Discussion

### 2.1 Design and Fabrication of PVDF‐Based Nanofibers

**Figure 1:**
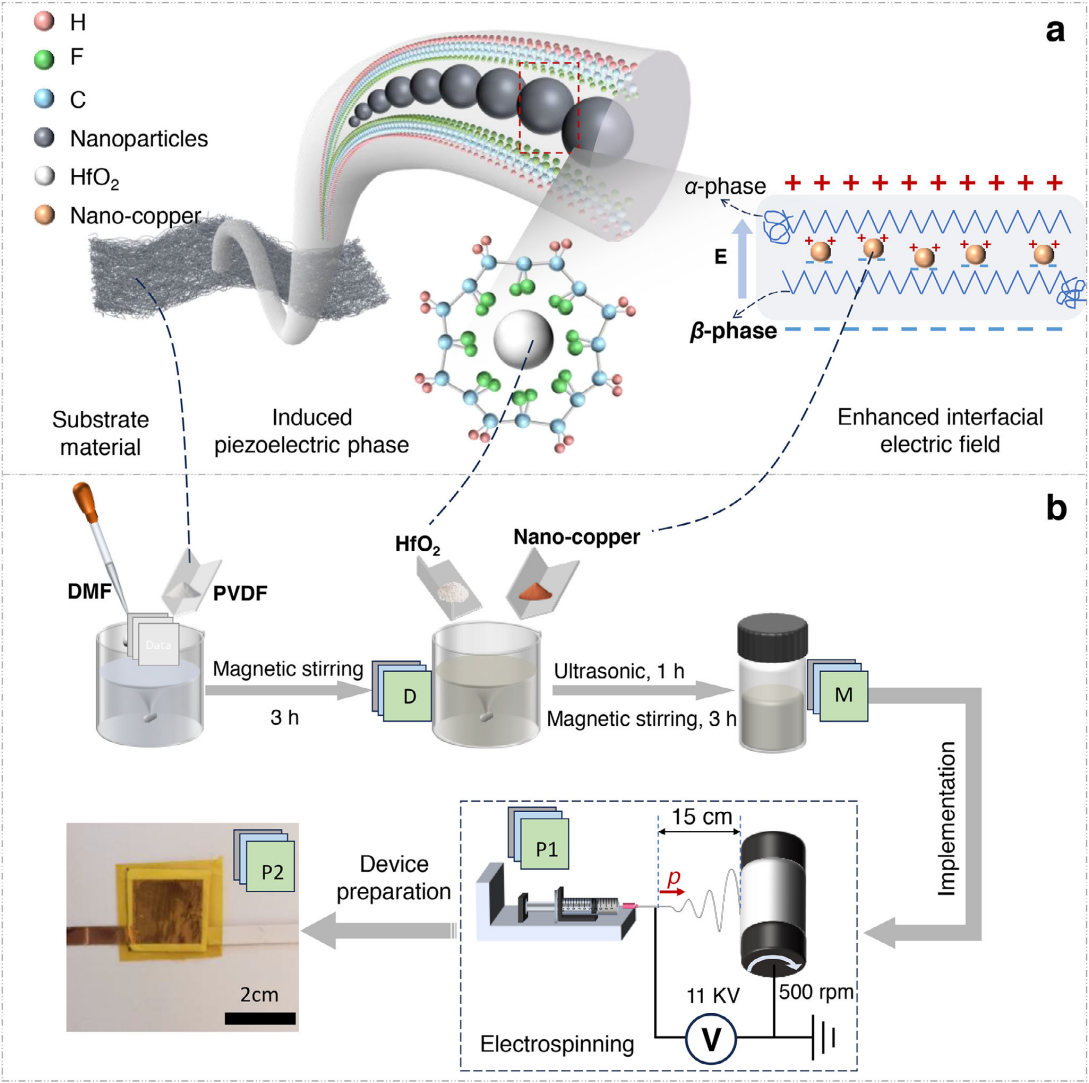
Rational design and fabrication of a piezo‐phototronic heterostructure. a) Conceptual diagram of the interface engineering within the PVDF composite, where HfO_2_ induces dipole alignment, and Cu nanoparticles create charge‐collection pathways. b) Diagram of the electrospinning process used to integrate the functional components into a unified nanofibrous architecture.

**Figure** **1**a illustrates the synergistic mechanism by which composite fillers enhance PVDF piezoelectricity through distinct yet complementary pathways. HfO_2_ doping induces *β*‐phase nucleation by electrostatic interaction−the high electronegativity of hafnium atoms preferentially coordinates with fluorine atoms in PVDF chains, forcing the alignment of CH_2_/CF_2_ dipoles into polar *β*‐conformation (Figure 1a, left).^[^ ^53^, ^56^ ^]^ The dual functionality of nano‐copper complements this structural templating effect: 1) as a heterogeneous nucleator that reduces crystallization energy barriers to boost overall crystallinity, and 2) as an electric field amplifier during electrospinning. The latter effect arises when surface charges on conductive Cu nanoparticles intensify the local field, generating enhanced electrostatic forces that mechanically align the PVDF chains into *β*‐phase domains.^[^ ^57^ ^]^ Furthermore, during electrospinning, the applied electric field induces surface charges of the nano‐copper, increasing the local electric field strength around the filler. This more potent electrostatic force further stretches the PVDF chains, promoting the formation of greater amounts of *β*‐phase (Figure 1a, right). The *β*‐phase ratio is directly related to piezoelectric performance; increasing it is crucial for improving piezoelectric properties.

The preparation of the composite nanofiber films is schematically illustrated in Figure 1b. A homogeneous precursor solution was prepared by dissolving PVDF, HfO_2_, and copper nanoparticles in DMF at optimized mass ratios. Electrospinning was subsequently conducted at room temperature, with detailed processing parameters provided in the Experimental Section. Under the applied high‐voltage field (≈11kV), the precursor solution from a Taylor cone at the spinneret tip generated a charged jet that underwent severe whipping and stretching due to electrostatic forces. This process yielded continuous, defect‐free nanofibers that were randomly deposited on the grounded collector, forming a nonwoven mat with homogeneous morphology.^[^ ^43^ ^]^

### 2.2 Morphological Characterization of PVDF‐Based Nanofibers

**Figure 2:**
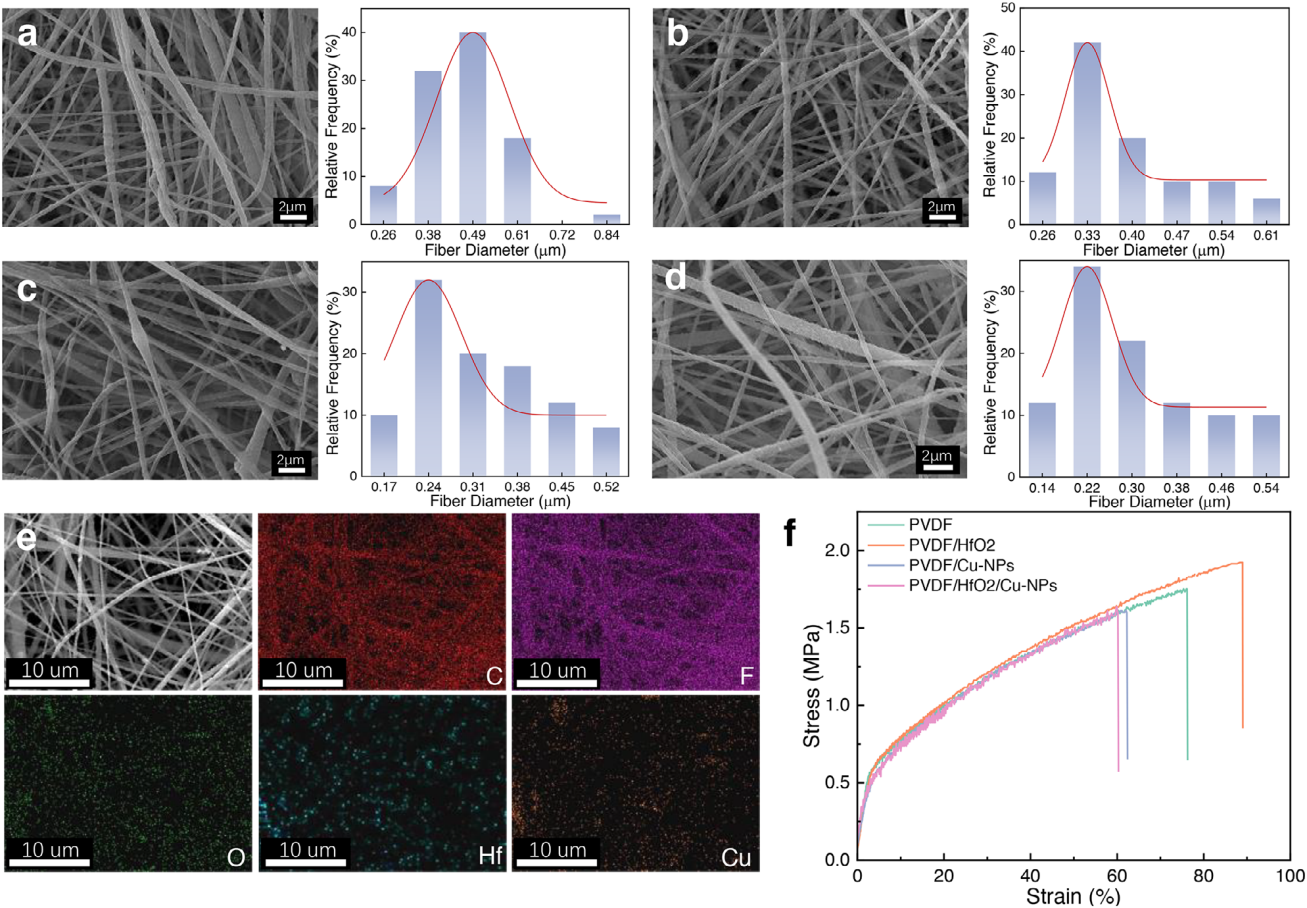
Microstructural characterization, elemental analysis, and mechanical properties of the electrospun composite nanofiber films. SEM images and corresponding fiber diameter distributions of: a) pristine PVDF, b) PVDF/HfO_2_, c) PVDF/nano‐copper, and d) PVDF/HfO_2_/nano‐Cu composite fibers. e) EDS spectrum verifying the co‐existence of C, F, O, Hf, and Cu elements in the ternary composite. f) Representative tensile stress‐strain curves comparing the mechanical performance of pure PVDF within the composite nanofiber films.

**Figure** **2**a–e and Figures S1 and S2 (Supporting Information) present representative scanning electron microscopy (SEM) images and corresponding fiber diameter distribution of pristine PVDF and its composite variants (PVDF/HfO_2_, PVDF/Cu, and PVDF/HfO_2_/Cu). All samples exhibited smooth, bead‐free fiber morphologies with uniform deposition characteristics. However, the introduction of functional fillers influenced both surface topography and dimensional uniformity. Pristine PVDF fibers displayed a smooth surface morphology with a narrow diameter distribution centered at 490 ± 110 nm (Figure 2a). Incorporation of HfO_2_ nanoparticles induced gradual surface roughening while simultaneously reducing average fiber diameters. At 1.2 wt.% HfO_2_ loading, fiber diameters decreased to is ≈330 ± 70 nm (Figure S1e,f, Supporting Information). Similarly, the addition of conductive copper nanoparticles (≤2 wt.%) produced fibers with textured surfaces and reduced diameters, reaching a minimum diameter of 240 ± 70 nm (Figure 2c). This refinement stems from enhanced solution conductivity, which intensifies the electric field‐induced stretching forces during jet elongation. However, exceeding the optimal filler concentration (3 wt.%) caused the fiber diameter enlargement to 400 nm (Figure S2e,f, Supporting Information), attributed to increased solution viscosity impeding efficient jet thinning, elevated surface tension counteracting electrostatic stretching forces, and nanoparticle aggregation inducing localized flow instabilities. These phenomena collectively demonstrate the delicate balance of solution properties and electrohydrodynamic processing parameters in determining final morphology. The observed diameter modulation suggests the potential to precisely tune nanofiber dimensions through controlled filler incorporation, which may subsequently influence electromechanical and optoelectronic performance.

Figure 2d presents a SEM image of the HfO_2_/nano‐copper composite fibers, revealing an ultra‐fine fiber morphology with diameters as small as 0.22 µm. Elemental mapping via energy dispersive spectroscopy (EDS) (Figure 2e) confirms the homogeneous distribution of C, F, and Hf throughout the fiber network. In contrast, Cu exhibits slightly non‐uniform dispersion − a phenomenon attributed to the high surface energy of nano‐copper particles, which promotes localized aggregation (Figure S3, Supporting Information). Notably, despite this tendency, no large‐scale Cu agglomeration was observed, demonstrating successful integration of both nanofillers within the polymer matrix. Shows the EDS images of the HfO_2_ and nano‐copper composite fiber films, where the distribution of Cu also exhibits slight non‐uniformity but without large‐scale aggregation. The EDS images further confirm the successful incorporation and good dispersion of HfO_2_ and nano‐copper. Additionally, the mechanical properties of the composite films show a distinct filler‐dependent behavior (Figure 2f). Compared to pure PVDF, samples incorporating HfO_2_ display enhanced elongation‐at‐break and tensile strength‐improvements that stem from two synergistic mechanisms: 1) increased surface roughness from well‐dispersed HfO_2_ nanoparticles, which promotes interfacial adhesion, and 2) effective stress distribution through the percolating HfO_2_ network, which impedes crack propagation.^[^ ^58^ ^]^ Conversely, films with higher nano‐copper loading exhibit diminished mechanical performance. This degradation results from two factors: the inherent hydrophobicity of copper nanoparticles that limits matrix compatibility, and their propensity for localized clustering, which disrupts stress transfer pathways.^[^ ^59^ ^]^ These findings highlight the critical trade‐off between functional fillers and structural integrity in nanocomposite design.

### 2.3 Multimodal Characterization of Nanofiber Architectures

**Figure 3:**
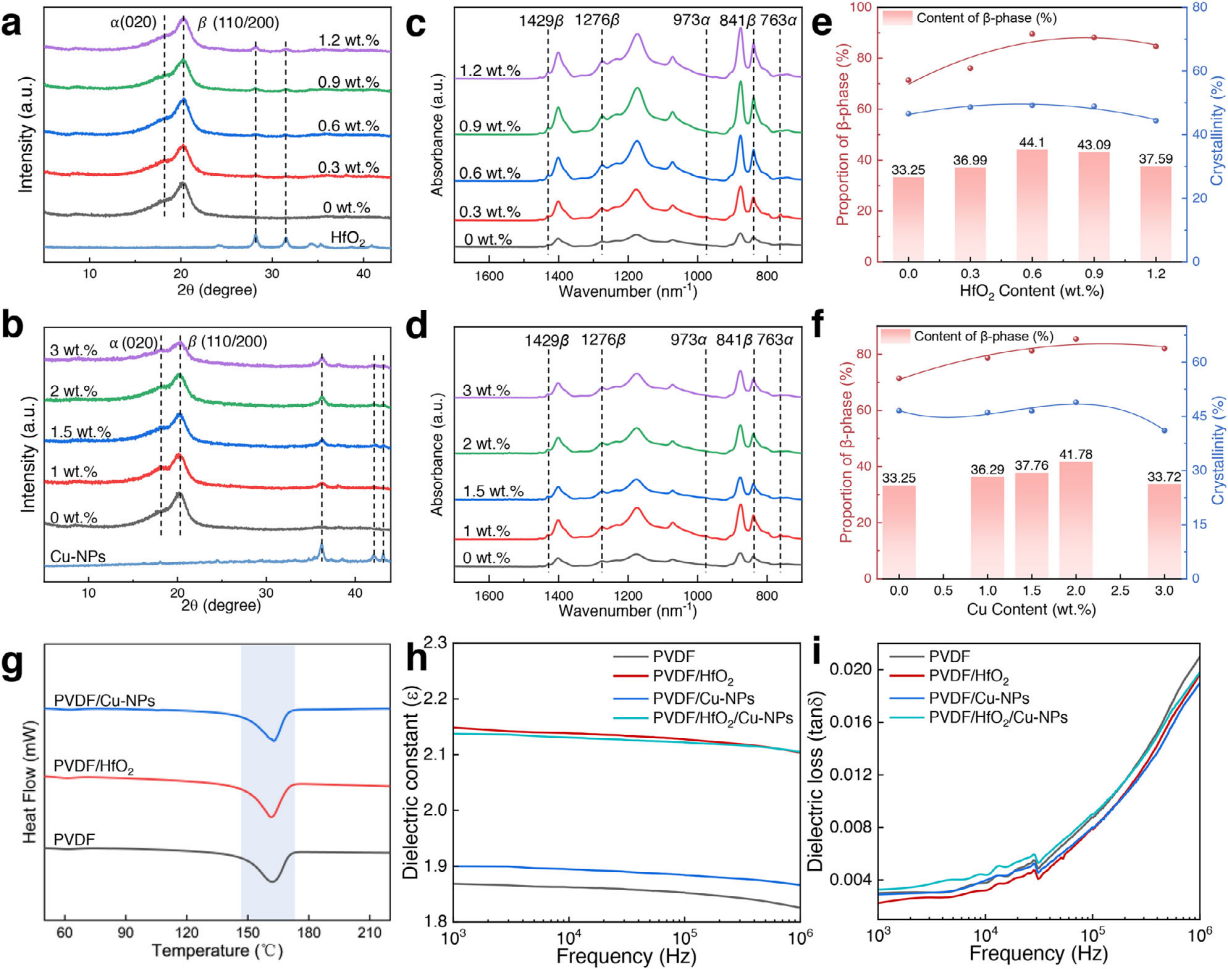
Structural and functional characterization of the composite nanofiber films after piezoelectric enhancement and intermolecular interactions. a) XRD patterns and c) FTIR spectra of PVDF and PVDF/HfO_2_ nanofiber films with varying HfO_2_ mass fractions. b) XRD patterns and d) FTIR spectra of PVDF and PVDF/nano‐copper nanofiber films with varying nano‐copper mass fractions. e, f) Quantitative analysis of crystallinity, *β*‐phase proportion, and content in PVDF/HfO_2_ and PVDF/nano‐copper fiber films. g) DSC thermograms comparing the thermal behavior of PVDF, PVDF/HfO_2,_ and PVDF/nano‐copper fiber films. h, i) Frequency‐dependent dielectric constant and dielectric loss for PVDF and its composite films (PVDF/HfO_2,_ PVDF/nano‐copper, and PVDF/HfO_2_/nano‐copper).

XRD analysis confirms filler incorporation and quantifies their impact on PVDF crystallinity (**Figure** **3**a,b). The diffraction peaks in the XRD patterns of fiber films with varying HfO_2_ content. The characteristic peaks of HfO_2_ appear at 28.4° and 34.5° (−111 and 111 planes of HfO_2_), respectively, and intensify with HfO_2_ loading, with the intensity of the HfO_2_ characteristic peaking at 1.2 wt.% (Figure 3a). Notably, the *β*‐phase diffraction (20.4°) maximizes at 0.6 wt.% HfO_2_, suggesting an optimal doping level for piezoelectric performance. For nano‐copper composites (Figure 3b), peaks at 36.2° (CuO), 42.3° (Cu_2_O), and 43.3° (Cu) confirm partial surface oxidation. This oxidation layer passivates copper nanoparticles, mitigating dielectric loss by limiting the formation of conductive networks and preventing charge leakage.^[^ ^60^ ^]^ FTIR spectra (Figure 3c,d) further corroborate the XRD results, with *β*‐phase vibrational modes (841, 1276, and 1429 cm^−1^) dominating over *α*‐phase signals (763 and 973 cm^−1^). The near‐absence of *α*‐phase peaks highlights the filler's efficacy in suppressing non‐polar crystallites. The content and crystallinity of fiber films with different fillers were analyzed using DSC thermograms (Figure 3g; Figure S4, Supporting Information), which reveal that the composites exhibit higher crystallinity than pure PVDF. According to the Lambert–Beer equation^[^ ^44^ ^]^:

(1) $$F\left(\beta \right)=\frac{A_{\beta }}{\left(\frac{K_{\beta }}{K_{\alpha }}\right)A_{\alpha }+A_{\beta }}\times 100\%$$

where *F*(β) is the relative content of the crystalline *β*‐phase; *K*_α_ and *K*_β_ are the absorption coefficients at 763 cm^−1^ (*α*‐phase) and 841 cm^−1^ (*β*‐phase), with values of 6.1×10^4^ and 7.7×10^4^ cm^2^ mol^−1^; *A*_α_ and *A*_β_ represent absorbances at 763 and 841 cm^−1^, respectively. Calculations show that the relative *β*‐phase content in pure PVDF nanofiber films is 71.41%. With the addition of HfO_2_, it reaches 89.54%, and with copper nanoparticles, it reaches 85.43%. This further confirms that the addition of fillers promotes *β*‐phase formation. Crystallinity is another crucial indicator of piezoelectric performance, which is measured by DSC. Figure 3g and Figure S4 (Supporting Information) show the DSC images of films with different fillers. The melting peaks of the films containing HfO_2_ range from 161.30 to 164.22 °C, while the films with nano‐copper have melting peaks between 161.84 and 163.13 °C, indicating no significant difference. It can be calculated by Equation (2)^[^ ^61^ ^]^:

(2) $$x_{c}=\frac{\Delta H_{m}}{\Delta H_{0}}\times 100\%$$

where *x_c_* is the crystallinity of the film, Δ*H_m_* is the melting enthalpy of the film with added fillers, and Δ*H*_0_ is the melting enthalpy of 100% crystalline PVDF (104.7 J g^−1^). Calculations show that the crystallinity of pure PVDF films is 46.56%, while the crystallinity of films containing HfO_2_ reaches a maximum of 49.25%, and movies containing nano‐copper reach a maximum of 48.91%. Figure 3e,f summarizes the *β*‐phase ratio and crystallinity of the composite films, with the *β*‐phase content in the composite fiber films calculated and presented (bar charts in Figure 3e,f). The data indicate that the *β*‐phase content of pure PVDF is 33.25%. As filler loading increases, this content initially rises and then declines. The content reaches a maximum of 44.1% when the HfO_2_ content is 0.6 wt.% and a maximum of 41.78% when the copper nanoparticle content is 2 wt.%. Characterization results indicate that doping with HfO_2_ and nano‐copper can enhance PVDF's piezoelectric properties.

Figure 3h,i shows the dielectric properties of pure PVDF, PVDF doped with 0.6 wt.% HfO_2_, PVDF doped with 2 wt.% nano‐copper, and PVDF simultaneously doped with 0.6 wt.% HfO_2_ and 2 wt.% nano‐copper. Figure 3h shows that the dielectric constant of films doped with HfO_2_ and nano‐copper is enhanced. This enhancement is attributed to the addition of appropriate fillers, which improve interfacial polarization with the polymer matrix. Furthermore, the dielectric loss of the composite fiber film remains nearly unchanged (Figure 3i), particularly for those containing nano‐copper, which do not show a substantial increase in dielectric loss. On the one hand, this is because the amount of nano‐copper added is moderate. Copper is an excellent conductor, and excessive amounts of conductive fillers can form conductive networks within the polymer matrix, leading to charge leakage and increased dielectric loss, which, in turn, reduces piezoelectric performance. On the other hand, some of the copper oxidizes (as confirmed by XRD patterns), which reduces the formation of conductive networks and decreases charge leakage. Therefore, doping with appropriate amounts of HfO_2_ and nano‐copper can enhance the dielectric and piezoelectric properties of the composite films.

### 2.4 Mechanical Sensing Performance and Durability

**Figure 4:**
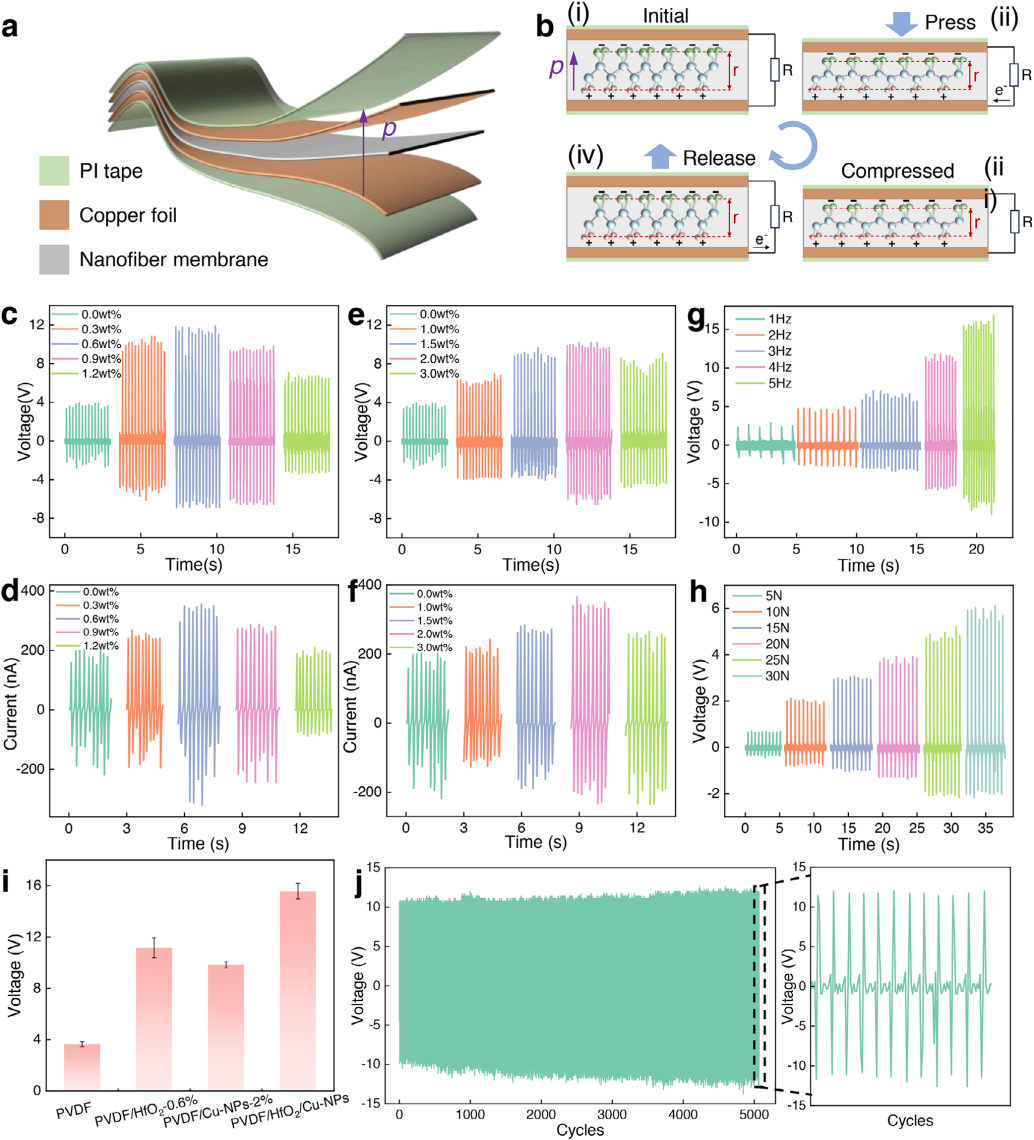
Piezoelectric performance evaluation of the nanofiber film‐based devices. a) Device structure and b) working mechanism schematics. Output characterization: c) voltage and d) current responses at varying HfO_2_ concentrations; e) voltage and f) current outputs with different nano‐copper contents. Performance analysis of PVDF/HfO_2_/nano‐copper: g) frequency‐dependent (1–5 Hz) open‐circuit voltage, of PVDF/HfO_2_/nano‐copper nanofiber film under pressing frequencies, h) pressing force response, and i) comparative voltage output among PVDF and its composites. j) Long‐term stability testing under cyclic loading (15 N, 5 Hz).

**Figure** **4**a illustrates the structural design of the piezoelectric device, consisting of a flexible fiber film sandwiched between two copper foil electrodes, with the entire assembly encapsulated in polyimide (PI) tape to isolate environmental interference and ensure stable piezoelectric performance. Figure 4b illustrates the operational mechanism: i) At equilibrium, the fiber film is electrically neutral. ii) Under applied mechanical pressure, the piezoelectric device, the fiber film undergoes compressive deformation, decreasing the distance (*r*) between fluorine and hydrogen atoms. This disturbance in charge distribution generates an internal electric field that drives charge flow through the external circuit via the top and bottom electrodes. iii, iv) Upon pressure release, the fiber film back to its original state, increasing *r* and producing a reverse voltage due to charge redistribution.

To investigate the effects of HfO_2_ and nano‐copper additives on piezoelectric performance, we fabricated a series of 20 mm × 20 mm piezoelectric devices (Figure 4a). Then, we compared the outputs of PVDF, PVDF/HfO_2_, and PVDF/nano‐copper composite fiber films under identical conditions (20 N, 5 Hz). The electrical responses of fiber films vs the varying doping concentrations of HfO_2_ and copper nanoparticles (Figure 4c–f). The electrical output of films under varying pressing frequencies (1–5 Hz) was also tested (Figures S5 and S6, Supporting Information). Pure PVDF fiber films generated a baseline output of 3.8 V and 190 nA. For HfO_2_ composites, both voltage and current initially increased with doping concentration, peaking at 0.6 wt.% HfO_2_ with 11.2 V (195% enhancement) and 355 nA (87% enhancement) (Figure 4c,d). Beyond this threshold, performance declined sharply due to filler aggregation and reduced *β*‐phase crystallinity. Filler aggregation, leading to uneven fiber diameters (Figure S1c–f, Supporting Information) and non‐uniform stress distribution with thinner fibers over‐deformed or fractured, while thicker fiber regions transferred stress inefficiently. Additionally, filler aggregation hinders *β*‐phase crystallization, thereby diminishing piezoelectric performance. Reduced *β*‐phase crystallinity results from excessive HfO_2_ disrupting PVDF chain alignment. For nano‐copper composites, the open‐circuit voltage peaked at 10 V (163% higher than PVDF) with 2 wt.% doping, while the current reached 350 nA (84% enhancement) (Figure 4e,f). Further doping led to aggregation‐induced defects (similar to those in HfO_2_ composites) and the formation of a conductive network (which short‐circuited piezoelectric charges, diminishing output). The performance trends align with material characterizations (e.g., crystallization kinetics and morphology). Optimal doping (0.6 wt.% HfO_2_ or 2 wt.% nano‐copper) maximizes the polar phase content, enhancing the piezoelectric response. Deviations from these concentrations introduce structural inhomogeneities or parasitic conductivity, degrading performance.

To explore the synergistic effect of dual‐phase doping, we fabricated a composite fiber film incorporating 0.6 wt.% HfO_2_ (the optimal concentration for HfO_2_) and 2 wt.% copper nanoparticles (the optimal concentration for nano‐Cu). Under identical loading conditions (20N, 5Hz), this dual‐doped device achieved a maximum output voltage of 15V —3.95× higher than pure PVDF (3.8 V) and surpassed that of single‐doped films (Figure 4g,i). Additionally, the PVDF/HfO_2_/nano‐copper composite fiber film demonstrated superior piezoelectric performance compared to single‐filler systems, generating output voltages 1.34 times that of PVDF/HfO_2_‐0.6 wt.% and 1.5 times that of PVDF/nanocopper‐2 wt.% (Figure 4i). Under controlled frequency testing (2 Hz), the composite fiber film exhibited robust pressure sensitivity, with its output increasing linearly from 0.6 V at 5 N to 6 V at 30 N (Figure 4h)—a response confirmed as piezoelectric in origin by the inverted signals observed during electrode reversal tests (Figure S7, Supporting Information). Durability assessments (15 N, 5 Hz) revealed stable operation for 5,000+ cycles, with an initial 10–15% output rise (attributed to interfacial charge accumulation) stabilizing to a consistent 10V output, demonstrating reliability under sustained high‐frequency mechanical stimulation (Figure 4j). The progressive voltage rise during initial cycles reflects a critical activation process, indicative of dynamic structural‐electrical coupling within the composite fiber network. This performance amplification arises from synergistic structural optimization and interfacial polarization stabilization mechanisms.

### 2.5 Piezoelectric Biomechanical Monitoring Platform

**Figure 5:**
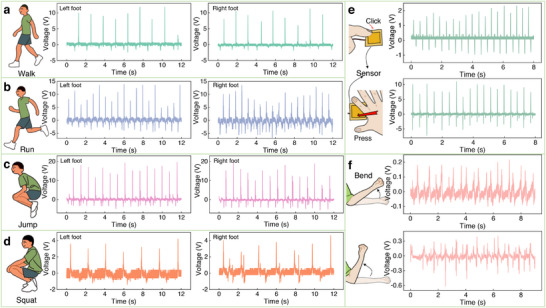
Applications of PVDF/HfO_2_/nano‐copper piezoelectric sensors in motion‐based energy harvesting and biomechanical sensing. Real‐time voltage output signals recorded from sensors attached to human toes during different motion states: a) walking, b) running, c) jumping, and d) squatting. e) Voltage response under hand‐induced mechanical stimuli (finger tapping and palm slapping). f) Real‐time voltage variation corresponding to different arm bending angles.

The optimized PVDF/HfO_2_/nano‐copper (0.6 wt.% HfO_2_, 2 wt.% nano‐copper) piezoelectric device serves as a high‐performance wearable sensor for human motion tracking, combining real‐time biomechanical monitoring with energy harvesting (**Figure** **5**; Figure S8, Movie S1, Supporting Information). When deployed on bilateral foot soles, the sensor distinguishes dynamic motion states (Figure 5a–d) through characteristic voltage signatures: a) Walking elicits periodic 10V signals with 0.62 s inter‐foot peak delay, reflecting low‐frequency ground contact forces (Figure 5a). b) Running intensifies mechanical inputs, shortening the inter‐foot delay to 0.35 s and boosting voltages to 14 V due to higher impact frequencies and amplitudes (Figure 5b). c) Jumping generates synchronized 20 V bilateral peaks, as simultaneous take‐off/landing concentrates body‐weight forces (Figure 5c). d) Squat‐standing introduces biomechanically driven signal polarity shifts, as evidenced by a 3.6 V toe‐dominated signal during upward thrust (center‐of‐mass elevation), contrasting with weaker heel‐predominant responses during descent, where partial sensor pressure release yields subtle reverse signals (Figure 5d; Figure S6d, Supporting Information). These state‐dependent electromechanical responses, spanning voltage magnitudes (10–20V), temporal patterns (0.35–0.62 s delays), and polarity variations validate the system's sensitivity to distributed foot‐pressure dynamics, enabling precise motion‐state identification.

Additionally, the PVDF/HfO_2_/nano‐copper piezoelectric sensor extends beyond foot motion tracking to precisely detect hand interactions and joint movements. As shown in Figure 5e, light finger taps generate a 2.3 V output, whereas a full‐palm slap, due to greater film deformation and contact area, produces a 10 V signal. The lower voltage compared to foot motions reflects reduced force application by hand. In addition to detecting output signals in pressing mode, this piezoelectric sensor can also detect bending‐mode signals. When mounted on an arm, the sensor captures voltage responses proportional to joint flexion (Figure 5f). Minor bending (e.g., 30°) yields 0.2 V. At the same time, deeper angles (e.g., 60°) increase output to 0.4 V. This angle‐dependent behavior arises from amplified film strain at larger deformations, though bending‐mode signals remain weaker than press‐mode outputs due to limited active material area. Collectively, these demonstrations highlight the dual functionality of piezoelectric devices as both versatile motion sensors and mechanical energy harvesters^[^ ^62^ ^]^, translating both compressive (e.g., footsteps, palm strikes) and tensile (e.g., joint bending) biomechanical inputs into quantifiable electrical signals.

### 2.6 Broadband Photoresponse and High‐Sensitivity Detection

Hafnium oxide (HfO_2_), a wide‐bandgap semiconductor, alters the optical properties of PVDF when incorporated into composite fibers. Optical tests were conducted on the composite fiber to determine whether doping HfO_2_ into PVDF could impart optical properties to the composite fiber. UV–vis absorption spectra (Figure S9c,d, Supporting Information) reveal that PVDF/HfO_2_ composite fibers exhibit a characteristic absorption peak at 225 nm and a calculated band gap (Eg) of 4.65 eV, closely matching pristine HfO_2_ (4.7 eV) (Figure S9a,b, Supporting Information). Notably, pure PVDF fibers lack such absorption features, confirming that the observed optical behavior originates from HfO_2_ doping.

**Figure 6:**
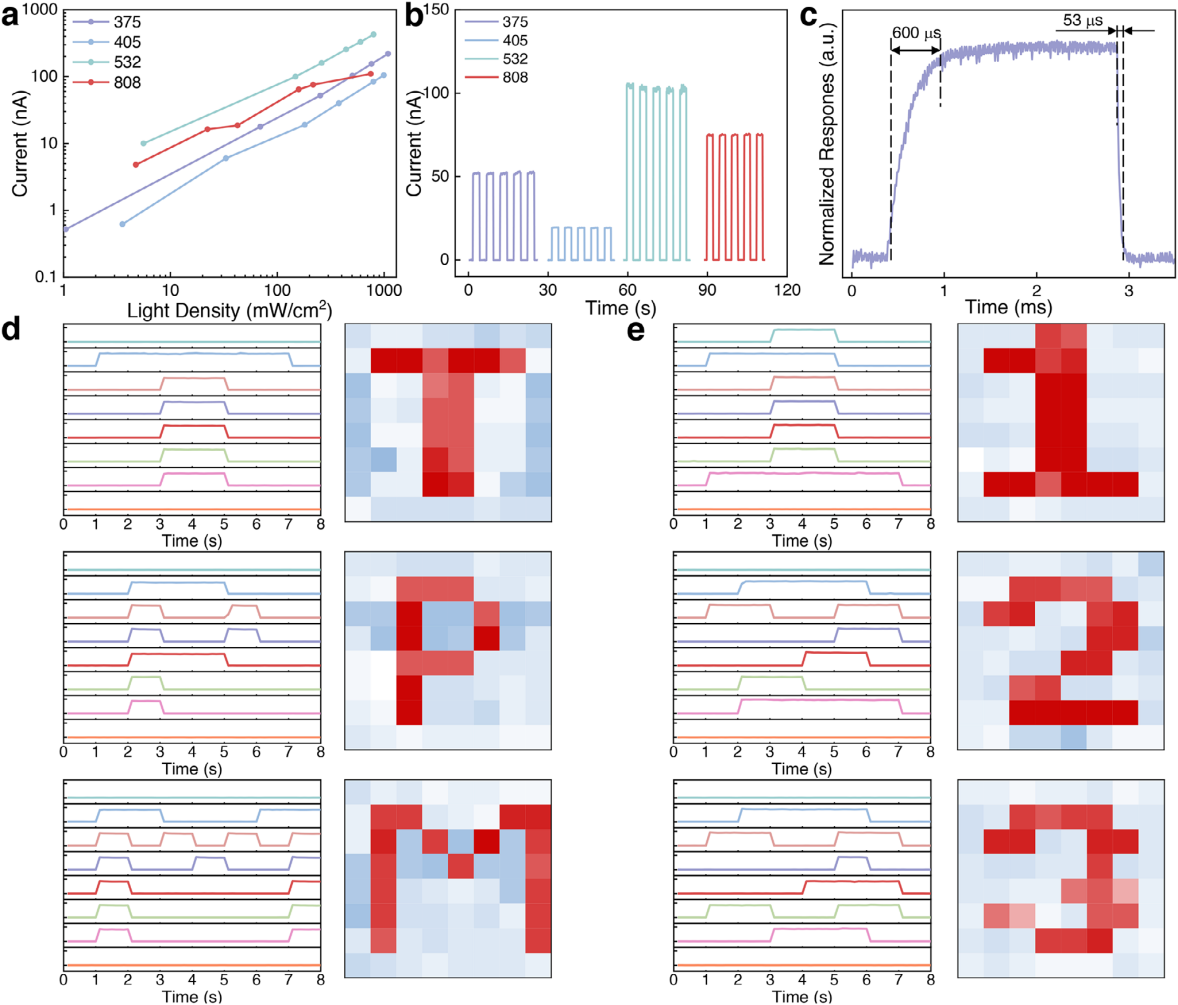
Optical output performance and imaging capability of PVDF/HfO_2_ composite nanofiber‐based devices. a) Wavelength‐ and power‐dependent photoelectric response. b) Spectral photoresponse at 0 V bias and 200 mW cm^−2^ irradiance. c) Single‐cycle photocurrent response during on/off illumination switching. d, e) Pattern recognition stability tests demonstrating imaging functionality.

To evaluate photoelectric performance, a fiber‐based photodetector (PD) was fabricated (Figure S10, Supporting Information). Under zero‐bias conditions, the device demonstrated broadband responsiveness across wavelengths from 375 to 808 nm (**Figure** **6**a), with photocurrent output showing a linear dependence on incident power over 375–532 nm. At a fixed power density (200 mW cm^−2^), the PD achieved maximal current output under 532 nm light (Figure 6b), indicative of wavelength‐dependent sensitivity. Further characterization quantified key figures of merit: responsivity (*R*) and specific detection rate (D*), both exhibiting strong correlations with light power (Figures S11–S14, Supporting Information). The repeatable on‐off cycling (2‐s intervals) and stable outputs (Movie S2) underscore the device's potential for low‐power, broadband optoelectronic applications spanning UV to near‐infrared detection. The responsivity of the PD can be calculated using Equation (3)^[^ ^63^ ^]^:

(3) $$R=\frac{I_{ph}}{P}$$

where *I_ph_* is the photocurrent and *P* is the incident light power. The responsivity of the PD decreases with increasing light power, reaching 5.67 mA W^−1^ under low‐intensity 532 nm light. Additionally, the specific detection rate is an essential metric for evaluating photodetector sensitivity. It can be calculated using Equation (4)^[^ ^63^ ^]^:

(4) $$D^{*}=\frac{R\sqrt{A}}{\sqrt{2eI_{d}}}$$

where *R* is responsivity, *A* is the effective area of the PD, *I_d_* is the current output of the PD in the absence of light, and *e* is the elementary electric charge. The photodetector demonstrates an inverse relationship between sensitivity and light intensity, achieving its peak specific detectivity of 4.14×10^11^ Jones under low‐intensity 375 nm illumination. This superior performance at minimal light levels confirms the device's sensitivity for detecting weak signals. Two key observations emerge from the spectral analysis: 532 nm illumination yields the highest photocurrent, suggesting optimal charge‐carrier generation under visible light, and 375 nm illumination provides the greatest detectivity, indicating superior signal‐to‐noise characteristics in the UV range. These results collectively verify that the electrospun PVDF/HfO_2_ composite fibers successfully retain and utilize the optical properties of HfO_2_. The material system demonstrates broadband responsiveness from UV to near‐infrared wavelengths, and the composite exhibits robust performance for UV‐selective detection applications. Based on these findings, all subsequent photodetector characterizations were conducted with 375 nm illumination to achieve optimal detectivity while maintaining consistency across measurements.

The temporal response characteristics of the PD were systematically evaluated through pulsed illumination tests at varying switching intervals (200, 500 µs, 1, and 5 ms). The device generates detectable photocurrent even at the shortest tested interval of 200 µs (Figure S15, Supporting Information), demonstrating its capability for high‐speed optical signal processing. In addition, the transition analysis (Figure 6c) shows that the current rises to 90% of saturation within 600 µs (ton) upon illumination, then decays to baseline in just 53 µs (toff) upon light cessation. This asymmetric yet rapid switching behavior demonstrates efficient charge carrier generation/collection mechanisms, accompanied by high‐speed recombination dynamics. Extended functionality tests confirm robust performance both in temporal stability and spatial resolution. Continuous irradiation measurements (Figure S16a, Supporting Information) exhibit negligible photocurrent decay over prolonged operation, indicating excellent photostability. When subjected to patterned illumination through mask‐defined obstacles (Figure S16b, Supporting Information), the device maintains both instantaneous response and consistent signal amplitude (Figure 6d,e). The combination of sub‐millisecond response and pattern‐invariant stability positions the electrospun PVDF/HfO_2_ composite as particularly suitable for dynamic optical sensing, high‐speed imaging, and reliable operation in variable lighting conditions.

### 2.7 Coupled Piezo‐Phototronic Effect and Energy Harvesting

**Figure 7:**
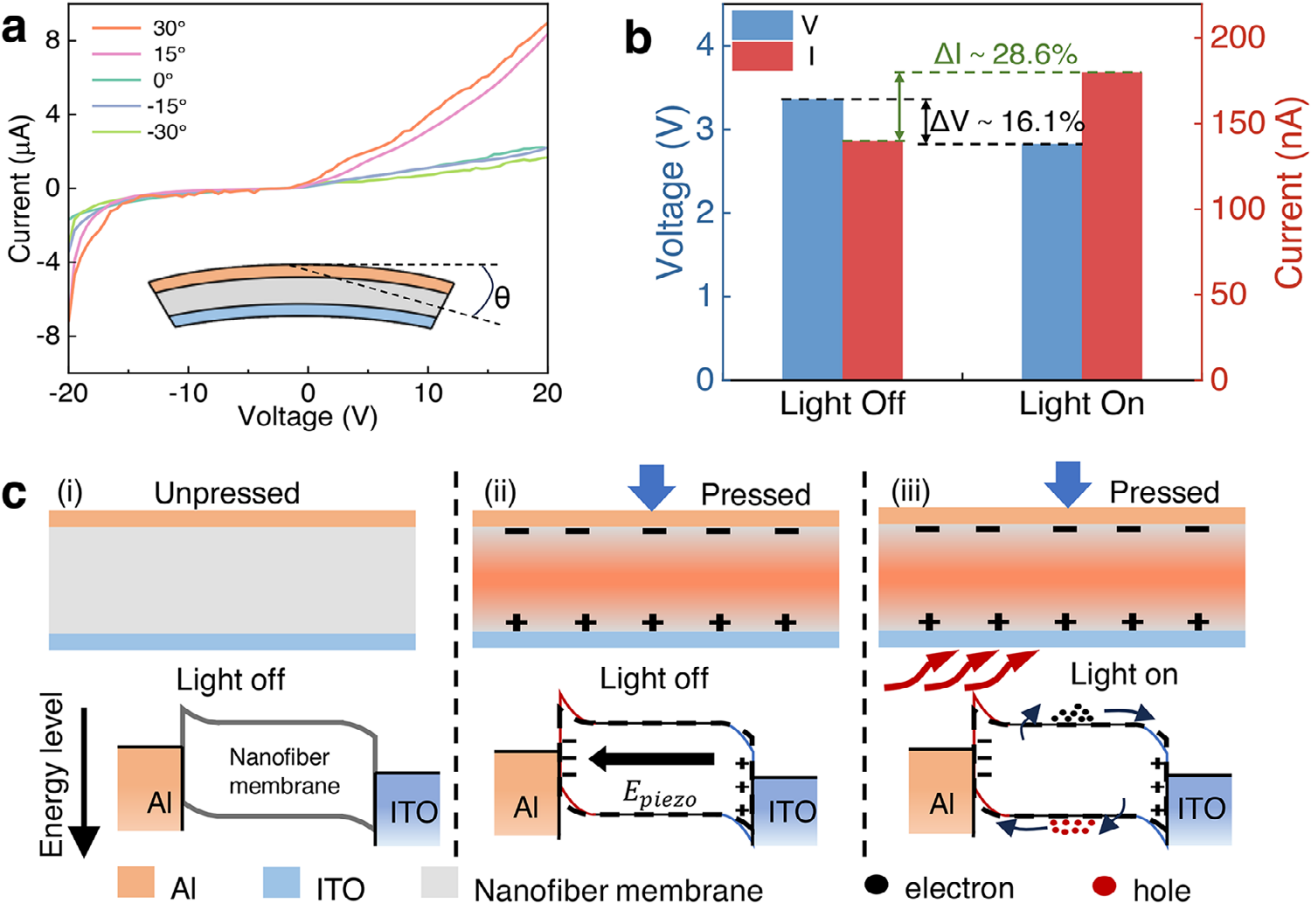
Piezo‐phototronic modulation in PVDF/HfO_2_ nanofiber films for multifunctional sensing applications. a) Current‐voltage characteristics under 375 nm illumination at varying bending angles. b) Voltage/current contrast between light and dark conditions. c) Energy band diagrams: (i) baseline (unpressed, dark), (ii) pressed‐modulated (dark), and (iii) pressure‐ and illumination‐activated states.

The piezo‐phototronic effect leverages the strain‐induced piezoelectric potential to modify the interfacial band alignment, thereby regulating device performance by enhancing carrier transport and separation efficiency. Our fabricated photodetector (PD) architecture consists of a trilayer structure: a transparent ITO electrode serving as the anode, a PVDF/HfO_2_ nanofiber film as the active piezoelectric‐semiconductor layer, and a copper foil cathode (Figure S18b, Supporting Information). The bending‐dependent electrical characteristics of the PVDF/HfO_2_‐based PD were systematically investigated over a ±20 V scanning bias range. The current–voltage (*I*–*V*) measurements (**Figure** **7**a) reveal a pronounced strain‐dependent modulation of photoelectric performance. Under convex deformation (positive bending angles), the PD exhibits enhanced photocurrent generation, suggesting improved charge‐transport efficiency. It was observed that the photocurrent of the PVDF/HfO_2_ film‐based PD increases with positive bending angles. Conversely, concave deformation (negative bending angles) suppresses photocurrent, demonstrating bidirectional strain‐responsiveness. This asymmetric response can be attributed to piezoelectric polarization in the PVDF matrix under mechanical stress, resulting from piezoelectric charge generation and Schottky barrier modulation. Applying strain to the film generates piezoelectric charges, which in turn modulate the Schottky barrier height^[^ ^64^ ^]^:

(5) $$\Delta \emptyset_{piezo}=-\frac{1}{2\varepsilon }\rho_{piezo}W_{piezo}^{2}$$

where ε is the dielectric constant of the material, ρ_*piezo*_ is the piezoelectric charge density at the contact interface, and *W_piezo_* is the width of the interfacial charge distribution. Under illumination, the Schottky barrier height can also be reduced^[^ ^20^ ^]^:

(6) $$\Delta \emptyset_{n}=-\frac{1}{2\varepsilon }\rho_{piezo}W_{piezo}^{2}-\frac{kT}{q}\ln\left(\frac{n_{0}+\Delta n}{n_{0}}\right)$$

where *k* is the Boltzmann constant, *T* is the temperature, *q* is the elementary charge, *n*_0_ is the electron density in the absence of light and external force, and Δ*n* is the increase in electron density under illumination. Thus, under the combined effect of light and mechanical force, the current density is given by:

(7) $$J_{n}=J_{no}\left(\frac{n_{0}+\Delta n}{n_{0}}\right)\exp\left(\frac{q}{kT}\frac{1}{2\varepsilon }\rho_{piezo}W_{piezo}^{2}\right)$$

where *J_no_* is the current density in the absence of illumination and external mechanical force, specifically, the PVDF/HfO_2_ film forms a Schottky contact with the underlying aluminum foil. Upon positive bending, the generated piezoelectric potential lowers the Schottky barrier height, facilitating electron‐hole pair separation and thereby enhancing the photocurrent output. Conversely, negative bending produces a negative piezoelectric potential, raising the barrier height and suppressing current generation. To evaluate the light‐dependent response, periodic pressure (1 Hz, 20 N) was applied under both illuminated and dark conditions (Figure 7b; Figure S17, Supporting Information). Under illumination, the piezoelectric voltage output decreased by 16.1%, while the current increase by 28.6% compared to dark conditions. Figure 7c provides a detailed illustration of the working mechanism: i) The baseline device structure and energy band diagram under dark and pressure‐free conditions. ii) Upon mechanical compression, piezoelectric polarization induces charge accumulation at the fiber film surface, causing an upward band shift on the left and a downward shift on the right. iii) Under illumination, photo‐generated charge carriers are separated more effectively due to the piezopotential, which retards electron‐hole recombination, thereby boosting the output.^[^ ^17^, ^64^, ^65^ ^]^ Simultaneously, charge carriers flowing enhance the local electric field, counteracting the net polarization and leading to attenuated voltage output.^[^ ^22^ ^]^

This composite film demonstrates dual functionality, combining superior piezoelectric performance with outstanding photoelectric responsiveness (see Table S1, Supporting Information for quantitative benchmarking against state‐of‐the‐art systems). Such synergistic properties position it as a leading candidate for next‐generation innovative wearable technologies and aerospace‐grade sensing solutions. As illustrated in Figure 7d, the sensor serves as a versatile platform for biomechanical and physiological monitoring. Its operational principles involve dynamic motion analysis, fall detection, elderly care, and tracking of rehabilitation progress. First, real‐time detection of stress variations during movements enables the precise characterization of gait dynamics, plantar pressure distribution, and joint kinematics in real‐time monitoring. Then, instant identification of balance‐loss incidents in mobility‐impaired individuals triggers emergency alerts to caregivers. Lastly, quantitative assessment of motor recovery in clinical settings provides physiotherapists with actionable data to optimize treatment protocols. Complementing its mechanical sensing capabilities, the embedded photodetection functionality enables continuous monitoring of ambient UV radiation intensity, providing science‐backed guidance for outdoor activity planning and skin health protection. The integration of piezoelectric and photoelectric effects enables a transformative multimodal sensing platform with broad applications in environmental monitoring, human health, and aerospace safety. In wearable and ecological systems, the optoelectronic function provides real‐time UV intensity tracking, which is critical for mitigating overexposure risks during outdoor activities. However, the sensor's true innovation lies in its dual‐mechanism structural health monitoring capability, which simultaneously detects mechanical impacts and microstructural defects through coupled piezoelectric and photoelectric responses. For aviation safety, this technology offers progress in tail strike prevention. When installed on aircraft tails, the piezoelectric component precisely quantifies impact forces during takeoff and landing. At the same time, the photoelectric module detects microfractures caused by scraping, enabling preemptive maintenance before catastrophic failure. This dual‐mode detection is equally vital in spacecraft and lunar exploration, where micrometeoroid impacts and extreme terrain constantly threaten structures. Piezoelectric arrays map collision energy distribution on spacecraft hulls and solar panels, while photoelectric circuits autonomously scan for surface cracks at stress‐critical joints. Such real‐time diagnostics are indispensable for orbital repair missions and adaptive rover navigation, ensuring mission continuity in unpredictable environments. A key advantage is that the multifunctional sensor features a self‐powering design, eliminating the need for an external energy source. Mechanical deformations and ambient light concurrently charge the sensor, reducing spacecraft preload mass compared to battery‐dependent alternatives. This energy autonomy, combined with ultrathin flexibility, allows seamless integration into curved aerospace structures and conformal wearables.

## 3 Conclusion

Using a facile, scalable electrospinning approach, we have developed a multifunctional PVDF‐based composite fiber film incorporating HfO_2_ and copper nanofillers that exhibit dual‐mode piezoelectric sensing capabilities. The composite structure demonstrates three key advantages. 1) The strategic incorporation of nanoscale dopants serves as effective nucleation sites that dramatically enhance the electroactive *β*‐phase from 33.25% to 44.1% in the PVDF matrix while simultaneously imparting broadband optical sensitivity spanning from near‐UV to near‐infrared wavelengths (300–1100 nm). 2) Under mechanical stimulation, the optimized composite fiber film generates outstanding piezoelectric outputs of 15 V and 355 nA at 5 Hz, representing substantial enhancements of 295% in voltage and 87% in current compared to pure PVDF fiber films, while maintaining remarkable stability through > 5,000 operation cycles‐a critical requirement for practical applications. 3) The material system demonstrates good optoelectronic performance, including high sensitivity (5.67 mA W^−1^) and detectivity (4.14×10^11^ Jones), ultrafast response characteristics (600 µs rise time, 53 µs decay time), and a significant piezo‐phototronic coupling effect (130% current enhancement under combined mechanical/optical stimulation. This work establishes a new paradigm in flexible sensor technology by achieving three notable progresses simultaneously: combining energy harvesting and optical detection in a single platform, enabling self‐powered operation through efficient piezoelectric conversion, and realizing synergistic performance enhancement through coupled physical effects. The straightforward fabrication method, coupled with these superior performance metrics, makes this technology particularly promising for next‐generation wearable health monitors and aerospace structural monitoring systems, where reliable, multifunctional sensing capabilities are paramount. These findings open up new possibilities for developing advanced, flexible electronics that integrate multiple sensing modalities while maintaining mechanical durability and energy autonomy — key requirements for practical implementation in real‐world applications. Future work will focus on further optimising the material composition and device architecture to enhance both sensitivity and environmental stability under various operational conditions.

## 4 Experimental Section

### Materials Preparation

Poly(vinylidene fluoride) (PVDF, Mw = 700 000 g mol^−1^) was procured from Arkema, while N, N‐dimethylformamide (DMF, AR grade) was obtained from Macklin. Hafnium oxide (HfO_2_, 99.9% purity) and nano‐copper (99.9%, 10–30 nm in diameter) were supplied by Aladdin and Meryer, respectively. All materials were used as received without further purification.

### PVDF‐Based Films Fabrication

PVDF powder was dissolved in DMF under continuous stirring to prepare a homogeneous 14% (w/v) solution. The mixture was maintained in a thermostatic water bath at 50 °C for 3 h until complete dissolution. HfO_2_ nanoparticles were subsequently introduced at varying concentrations (0.3−1.2 wt.%) into the PVDF solution, followed by ultrasonication (1 h) to ensure uniform dispersion. The mixture was then stirred for an additional 3 h at 50 °C, yielding a stable electrospinning precursor. A similar process was employed for PVDF/nanocopper composite solutions, where copper nanoparticles (1−3 wt.%) were dispersed under identical conditions. The resulting precursor solution was loaded into a 10 mL syringe fitted with a 20G stainless steel needle and electrospun. The electrospinning process parameters were strictly controlled as follows: ambient conditions of 25 ± 2 °C and 40% relative humidity, 11 kV applied voltage, 0.8 mL h^−1^ flow rate, 15 cm needle‐to‐collector distance, and 5 h deposition duration.

### Fabrication of Sensing Devices

The as‐prepared nanofiber films were sectioned into square specimens (20 mm × 20 mm), and copper foil electrodes (≈50 µm thick) were affixed to both surfaces. Conductive copper tape was applied to establish robust electrical contact between the electrodes and external measurement circuitry. To ensure environmental stability and minimize mechanical degradation, the assembled device was encapsulated with polyimide (PI) insulating tape, yielding a fully packaged piezoelectric sensor with enhanced operational reliability. For photodetector construction, the PVDF/HfO_2_ composite fibers were directly electrospun onto a pre‐patterned silicon substrate bearing interdigitated gold electrodes. The nanofibers were strategically deposited to bridge adjacent electrodes, thereby ensuring optimal charge‐transport pathways. The gold microelectrodes simultaneously functioned as both the active sensing interface and external electrical contacts, eliminating the need for secondary metallization while maintaining spectral sensitivity. The device used a lateral electrode structure, and its piezoelectric response primarily operates in the d33 mode. When external mechanical stress was applied along this thickness direction, the generated piezoelectric potential was parallel to the polarization direction, thereby producing the strongest output signal between the upper and lower electrodes.

### Materials Characterization

Field‐mission scanning electron microscopy (FE‐SEM, JSM‐7800F) was employed for comprehensive microstructural evaluation and elemental characterization. X‐ray diffraction (XRD, PANalytical X'Pert Pro) analysis was conducted to determine crystallographic properties, while Fourier‐transform infrared spectroscopy (FTIR, Nicolet iS50) provided detailed surface chemical composition analysis.

### Data Acquisition

Tensile properties of the nanofiber films were quantified using a high‐precision universal tensile system (DLS‐07 PC electronic tensile tester) under controlled ambient conditions. Optical absorption characteristics were acquired using a UV–vis spectrophotometer (Shimadzu UV2600) synchronized with a pulse generator. The voltage output performance of the piezoelectric devices was measured with an oscilloscope (DS1202Z‐E, RIGOL Instruments), and the current output of the photodetector was measured using an electrometer (Keithley 2400). The device's response time was measured with an oscilloscope with a pulse source. For photoresponse studies, wavelength‐specific laser diodes (375, 405, 532, and 808 nm) provided calibrated illumination, with optical power density meticulously controlled and monitored using a precision optical power meter (FieldBest).

## Conflict of Interest

The authors declare no conflict of interest.

## Author Contributions

J.G. and Q.P. contributed equally to this work. J.G and Q.P are co‐first authors. Y.Z. and Z.Y. conceived and coordinated the project. J.G. designed and fabricated the sensors. J.G. and Q.P. performed the series performance tests. X.Z., R.Z., and X.S. assisted in the construction of the optical testing sketches for photonic performance. Y.Z. and Z.M. assisted in data analysis. J.G., Q.P., X.S., B.L. and Y.Z. assisted in the device fabrication and piezoelectric characterization. Q.P. and Y.Z. assisted in morphological characterization and piezoelectric biomechanical monitoring. J.G. and Y.Z. assisted in multimodal characterization. Z.M. and Z.Y. assisted in the piezo‐phototronic effect analysis. J.G. and Q.P. wrote the draft. X.Z., Y.Z., Z.M., X.S., R.Z., B.L., Y.Z., and Z.Y. reviewed and edited the manuscript.

## Supporting information



Supporting Information

Supporting movieS1

Supporting movieS2

## Data Availability

The data that support the findings of this study are available from the corresponding author upon reasonable request.
