# Supplementary material for: Piezo‐Phototronic PVDF/HfO2/Nano‐Cu Heterostructured Thin Film for Flexible Self‐Powered Multimodal Sensing
Source: Adv Sci (Weinh). 2025 Dec 7;13(11):e18913. doi: 10.1002/advs.202518913 (PMC12931236; doi:10.1002/advs.202518913)
Supplement: Supplementary file 1 — Supporting Information [file ADVS-13-e18913-s003.pdf]

Supporting Information

Piezo-phototronic effects of PVDF/HfO_2/Nano-Cu heterostructured film for flexible self-powered multimodal sensing

Jiawei Gu[1#], Qiongle Peng[2#], Xuanqi Zhong[1], Yi Zheng[3], Zhiqiang Ma[4], Xiaoxian Song[1], Ruihuan Zhang[1], Duorui Yang[1], Bao Liu[5], Yanhu Zhang[1*], Zhengbao Yang[3*]

1. School of Mechanical Engineering, Jiangsu University, No. 301 Xuefu Road, Zhenjiang 212013, China

2. Department of Blood Transfusion, Affiliated Hospital of Jiangsu University, No. 438 Jiefang Road, Zhenjiang 212000, China

3. Department of Mechanical and Aerospace Engineering, Hong Kong University of Science & Technology, Hong Kong SAR 999077, China

4. Department of Biomedical Engineering, City University of Hong Kong, 83 Tat Chee Avenue, Kowloon, Hong Kong, 999077, China

5. Automotive Engineering Research Institute, Jiangsu University, No. 301 Xuefu Road, Zhenjiang 212013, China

[#] Jiawei Gu and Qiongle Peng contributed equally.

*Corresponding authors: zhyh@ujs.edu.cn (Zhang) and zbyang@ust.hk (Yang).

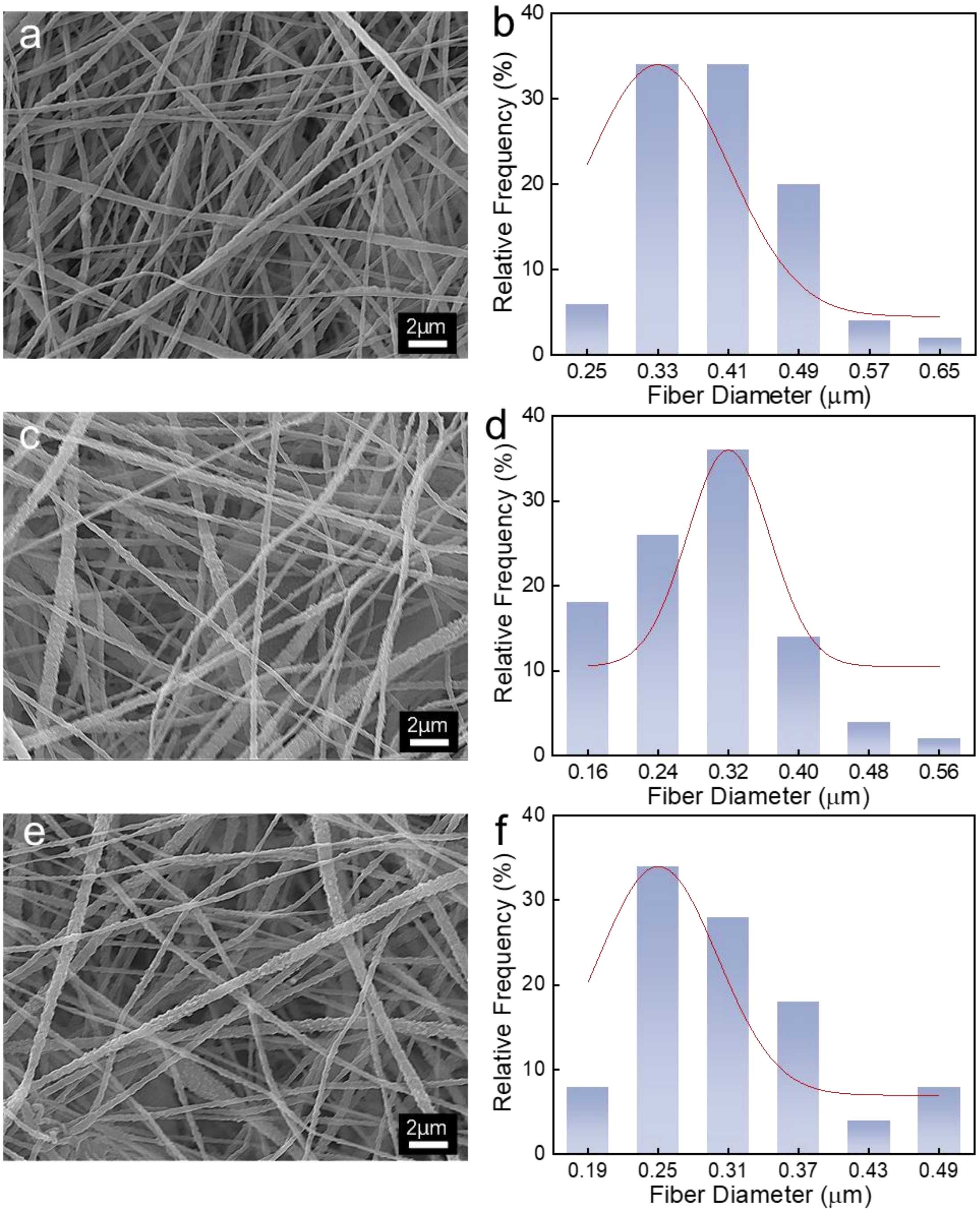

Fig. S1 Effect of HfO_2 doping concentration on the morphology of electrospun PVDF nanofibers. Representative SEM images (a, c, e) and their corresponding fiber diameter distributions (b, d, f) for HfO_2 concentrations of 0.3 wt.%, 0.9 wt.%, and 1.2 wt.%, respectively. The distributions (histograms) are fitted with Gaussian curves (solid red lines), demonstrating the evolution of average fiber diameter and morphological homogeneity with filler content.

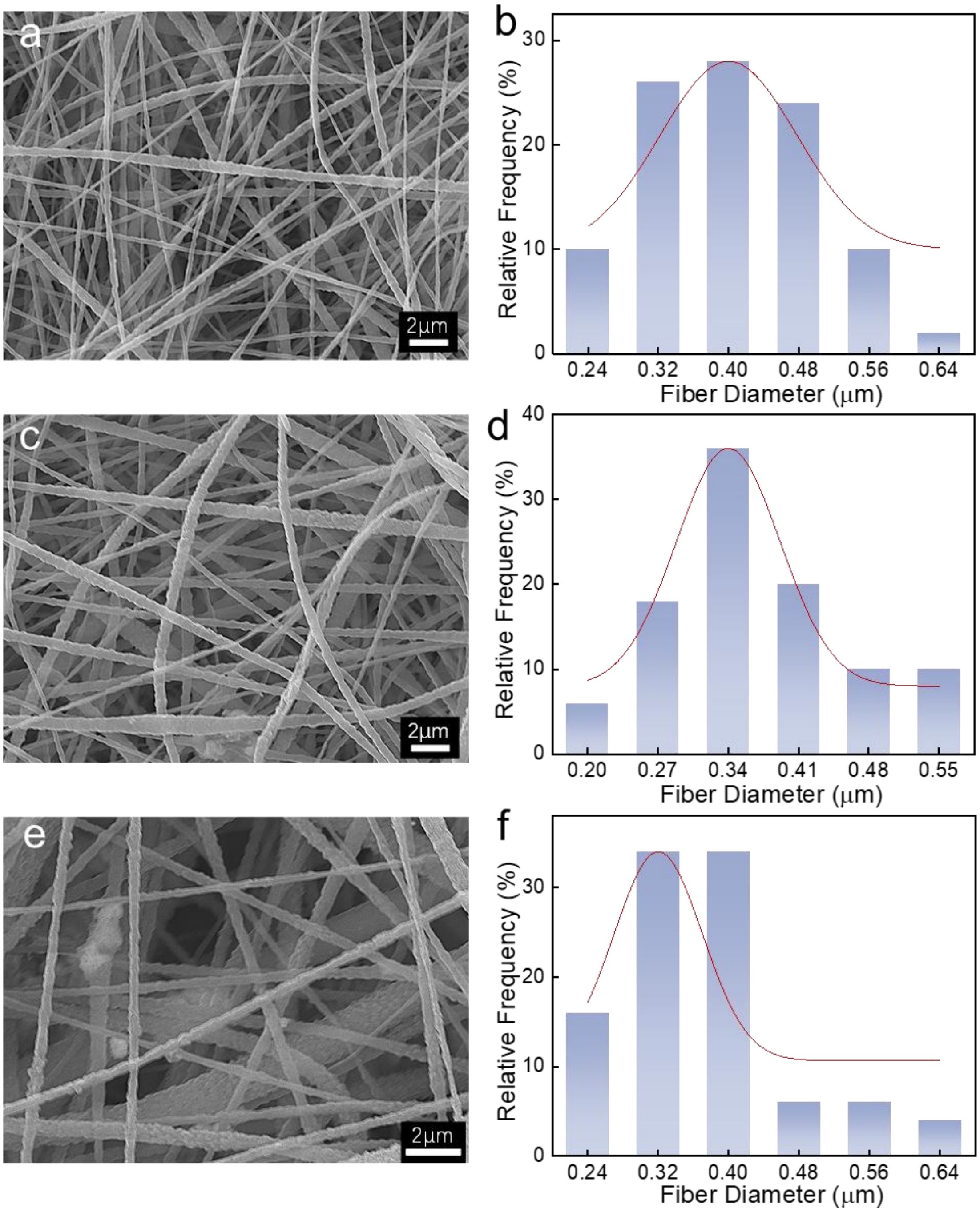

Fig. S2 Evolution of morphology in nano-copper-doped PVDF nanofibers. SEM images and fiber diameter distributions for doping concentrations of (a, b) 1.0 wt.%, (c, d) 1.5 wt.%, and (e, f) 3.0 wt.%. The morphology transitions from uniform fibers at lower concentrations to a structure with evident nanoparticle clusters and increased fiber diameter at 3.0 wt.%, indicating aggregation that can compromise electrospinning quality and composite properties.

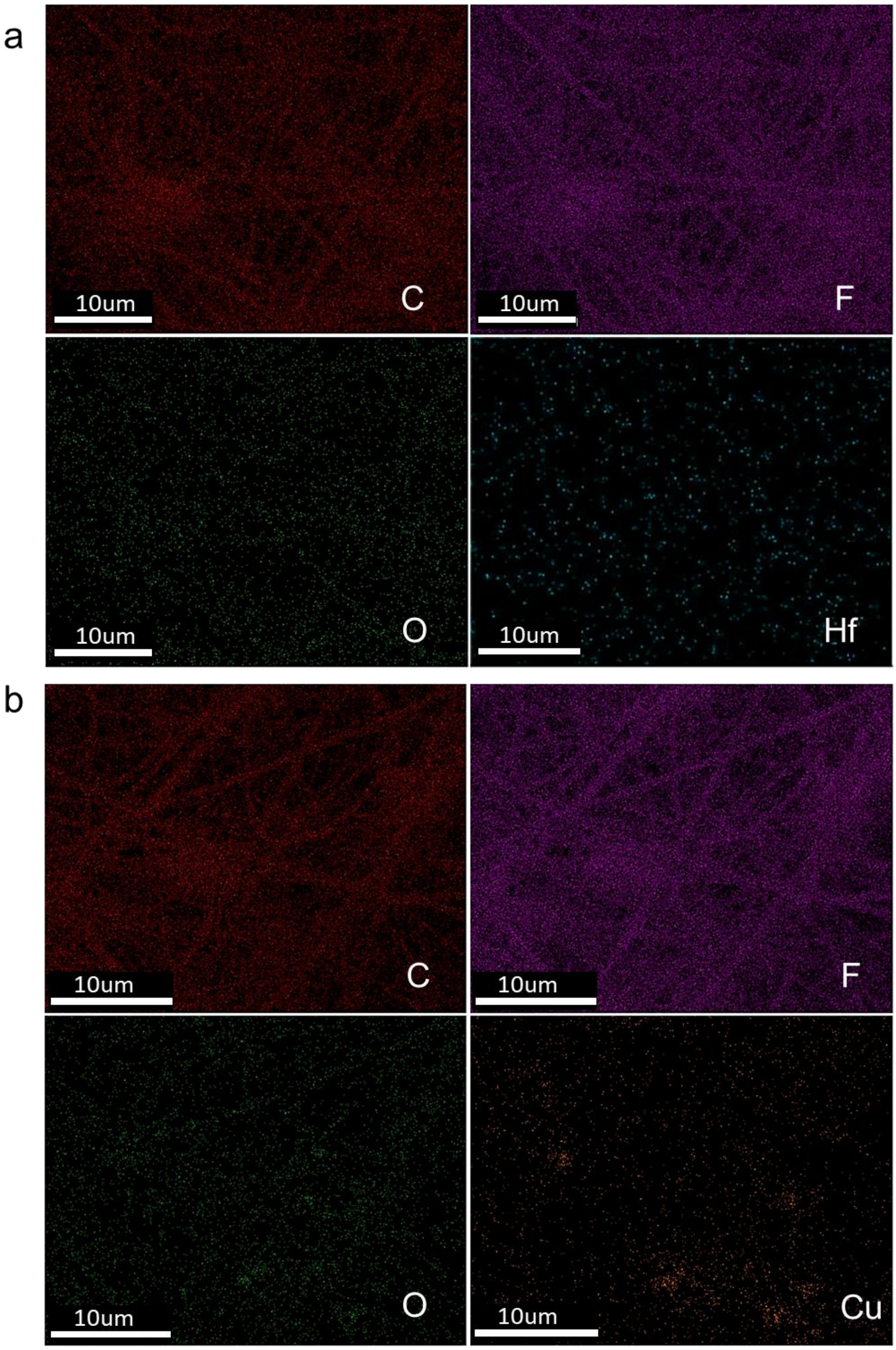

Fig. S3. Comparative EDS elemental mapping of the composite nanofibers. (a) PVDF/HfO_2 (0.6 wt.%) composite, showing a homogeneous spatial distribution of carbon (C), fluorine (F), oxygen (O), and hafnium (Hf) elements, which confirms the uniform dispersion of HfO_2 nanoparticles. (b) PVDF/nano-copper (2.0 wt.%) composite, revealing the distribution of C, F, O, and copper (Cu). While the polymer matrix elements (C, F) are uniform, the Cu signals exhibit localized intensity variations (clusters), indicating a less uniform dispersion compared to HfO_2.

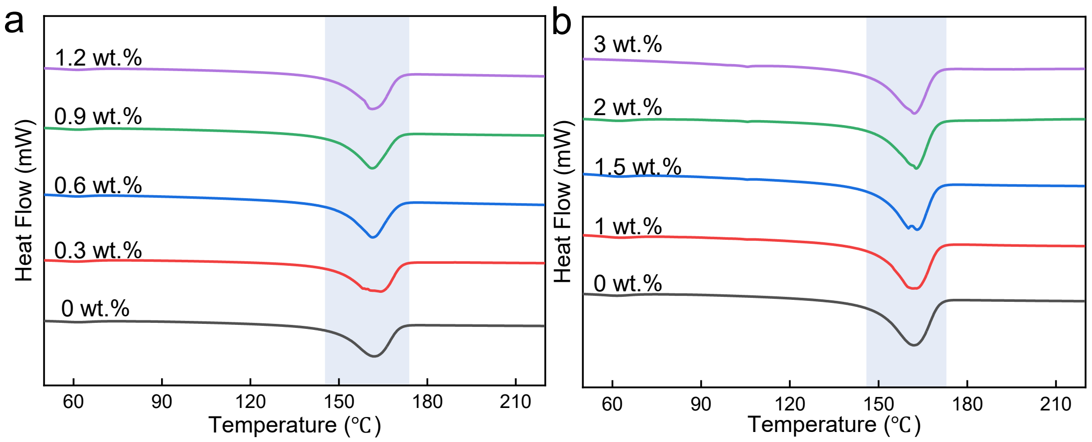

Fig. S4. DSC thermograms acquired under a nitrogen atmosphere during the second heating cycle, showing the melting behavior of: (a) PVDF/HfO_2 and (b) PVDF/nano-copper composite nanofibers as a function of filler mass fraction. The shifts in melting temperature (T_m) and changes in melting enthalpy reflect the influence of fillers on the crystallinity and thermal stability of the PVDF matrix.

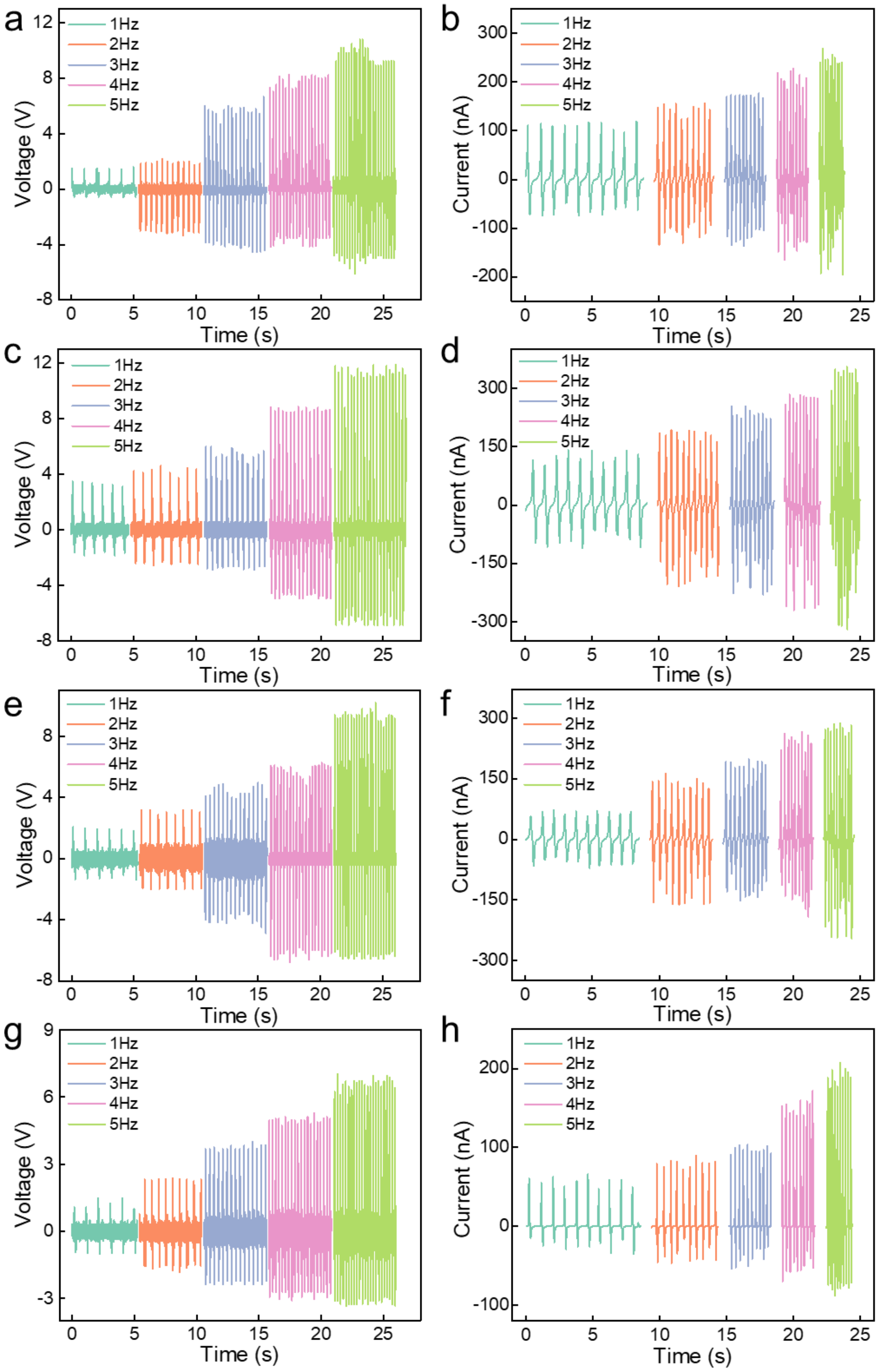

Fig. S5 Piezoelectric output performance of PVDF-based nanofibers with increasing HfO_2 doping concentrations. The open-circuit voltage (left panels) and short-circuit current (right panels) are shown for HfO_2 concentrations of: (a, b) 0.3 wt.%, (c, d) 0.6 wt.%, (e, f) 0.9 wt.%, and (g, h) 1.2 wt.%, revealing a trend of performance enhancement with increasing filler content up to an optimal level.

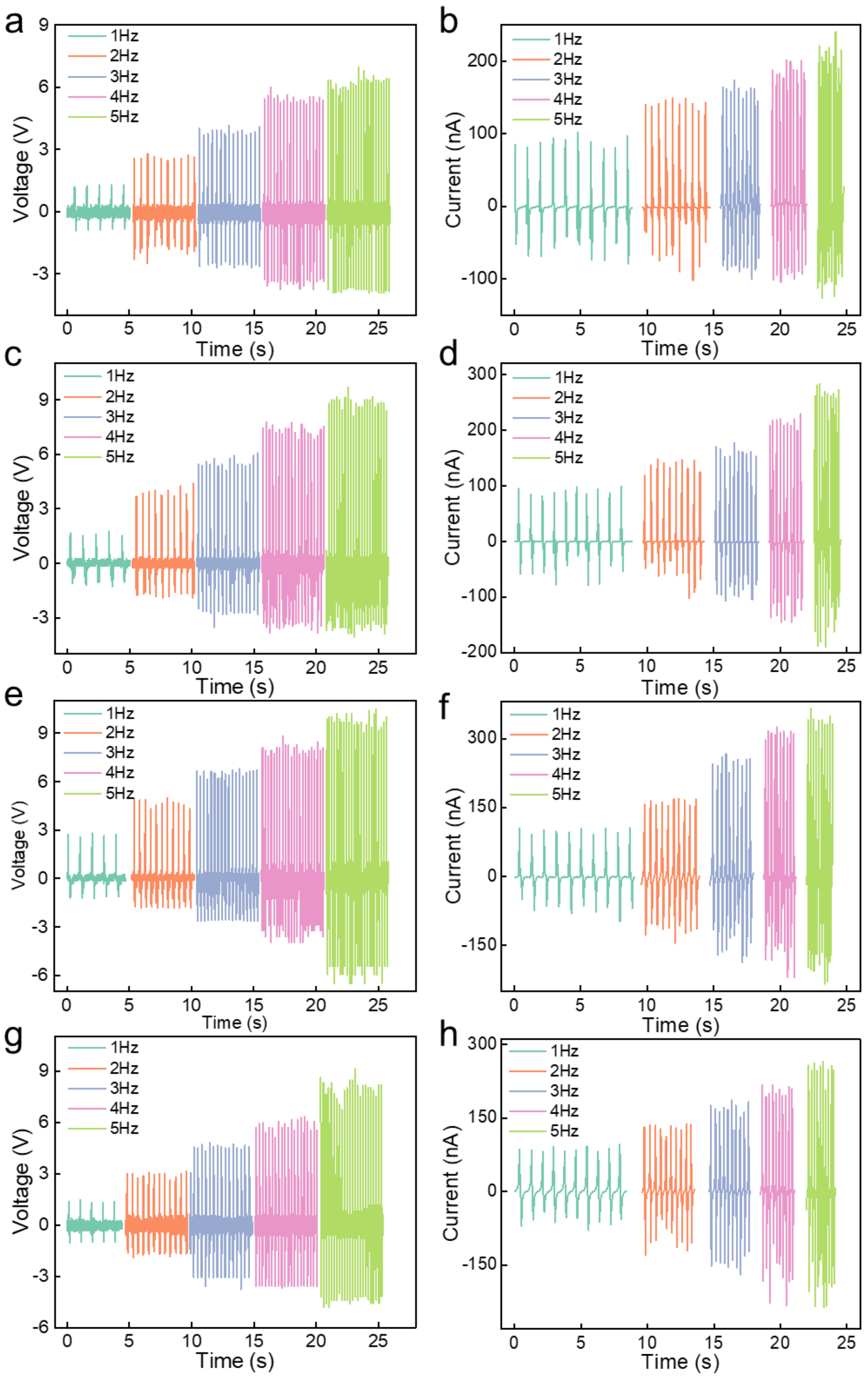

Fig. S6. Piezoelectric output performance of PVDF nanofibers with varying nano-copper content under the increasing loading frequency. Open-circuit voltage (left panels) and short-circuit current (right panels) are shown for nano-copper concentrations of: (a,b) 1.0 wt.%, (c,d) 1.5 wt.%, (e,f) 2.0 wt.%, and (g,h) 3.0 wt.%. The outputs demonstrate a clear dependence on filler concentration, with optimal performance observed at intermediate loadings before aggregation effects become dominant at higher concentrations.

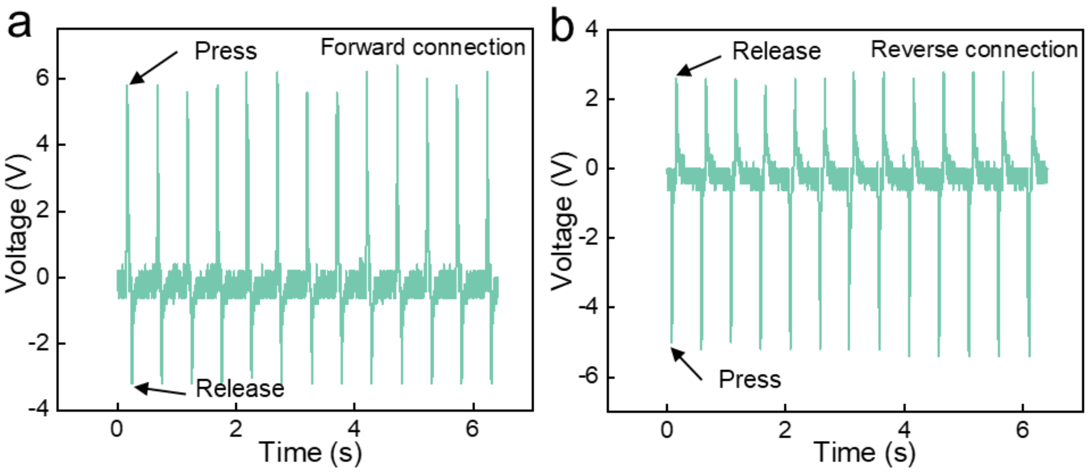

Fig. S7. Output voltage signals of piezoelectric electronics demonstrating polarity inversion, a hallmark signature of piezoelectricity. under (a) forward and (b) reverse electrode connections. (a) With a forward electrode connection, mechanical pressing yields a positive voltage but releasing produces a negative response. (b) Simply reversing the electrode connections inverts the signal polarity, as expected for a genuine piezoelectric source. This clear and consistent polarity reversal upon connection swap provides irrefutable evidence that the electrical output is a direct result of piezoelectric effect within the PVDF-based composite film material.

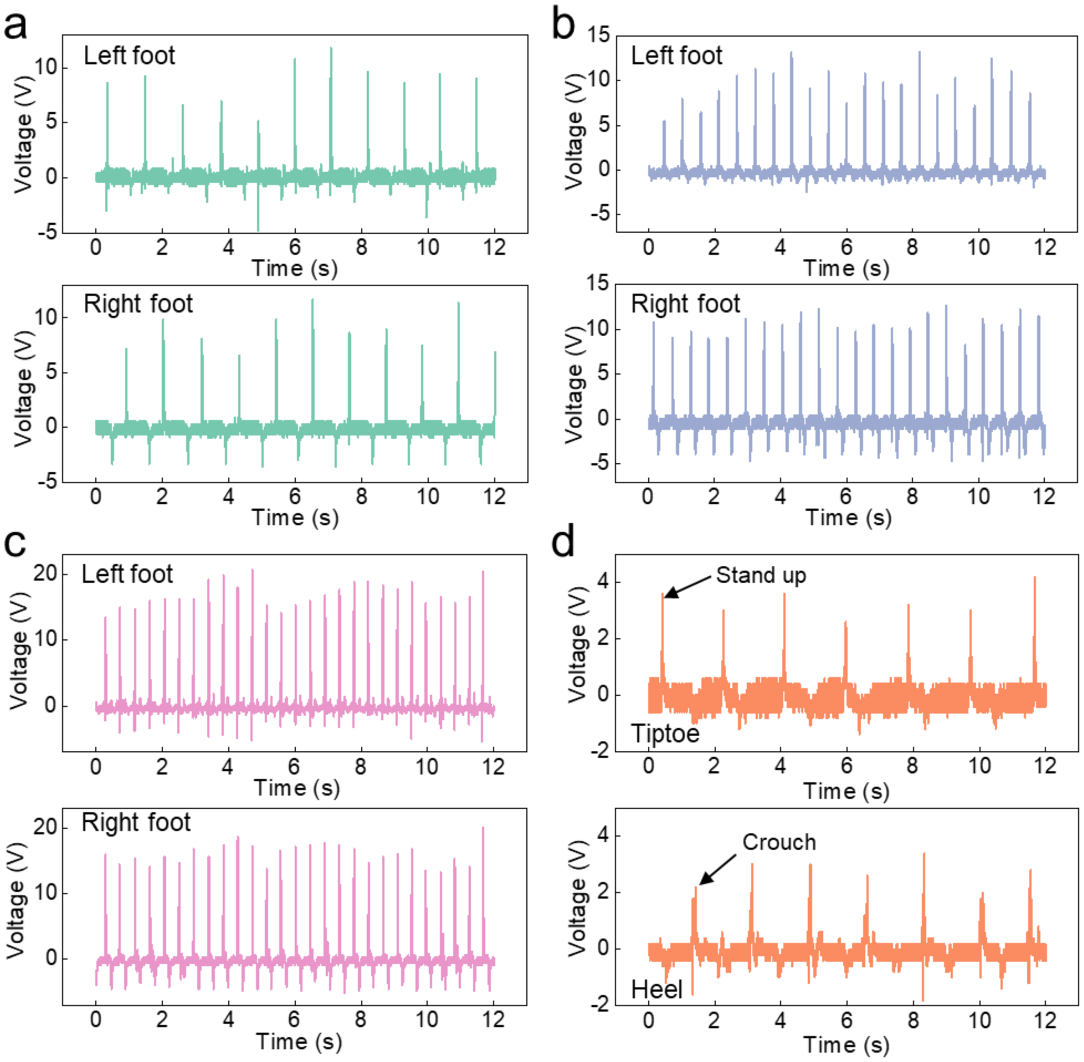

Fig. S8 In vivo monitoring of biomechanical motions using a wearable piezoelectric sensor. Real-time voltage signals were recorded from the piezoelectric sensors placed on (a-c) the heel during the rhythmic activities of (a) walking, (b) running, and (c) jumping, respectively. (d) Simultaneous signals from sensors on both the toe and heel during a squatting cycle, capturing the distinct force profiles at different parts of the foot. The distinct signal profiles demonstrate the sensor's capability for detailed motion analysis.

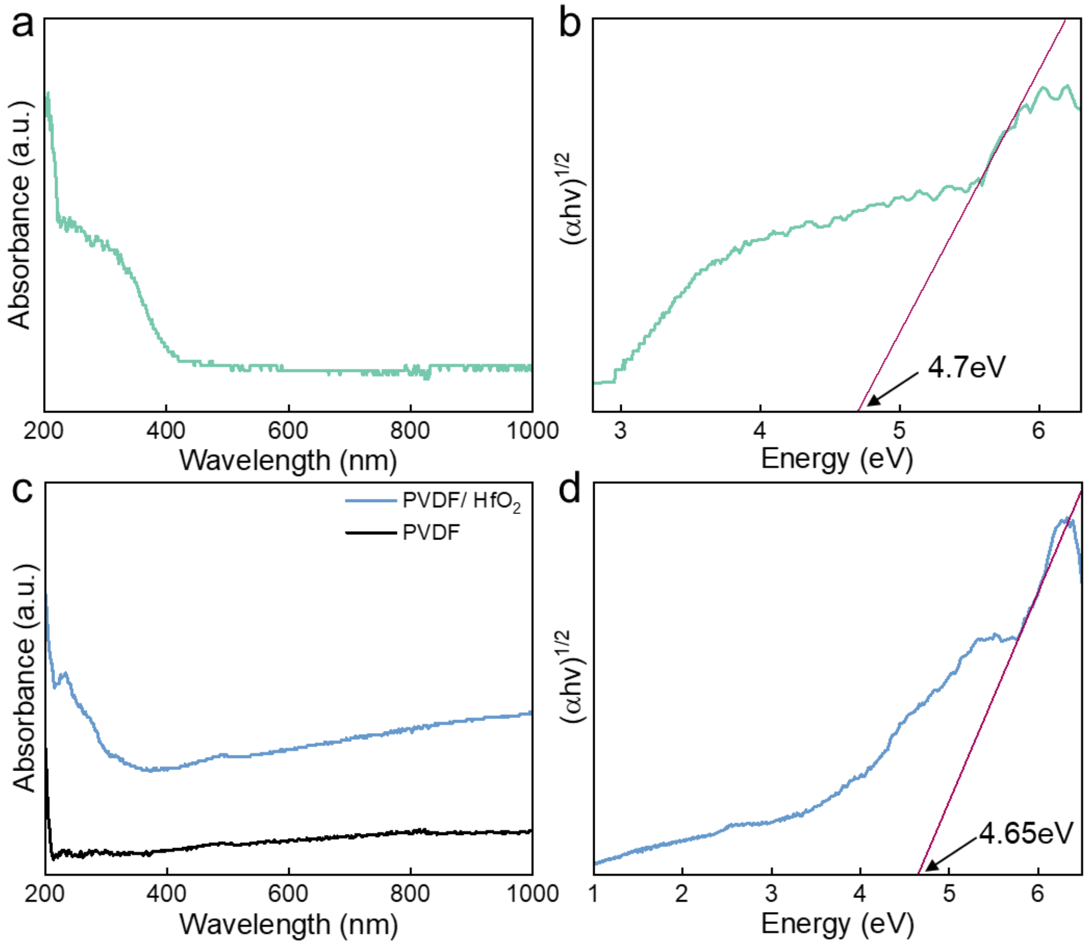

Fig. S9. Systematic optical characterization and bandgap analysis. (a) UV-vis absorption spectra of pristine HfO_2 particles and (c) PVDF and PVDF/HfO_2 composite nanofibers in the range 250-1000 nm. Corresponding Tauc plots derived from the absorption data demonstrate the indirect bandgap transition for (b) HfO_2 particles and (d) PVDF/HfO_2 nanofibers, confirming the successful integration of HfO_2 and its contribution to the composite's optical properties.

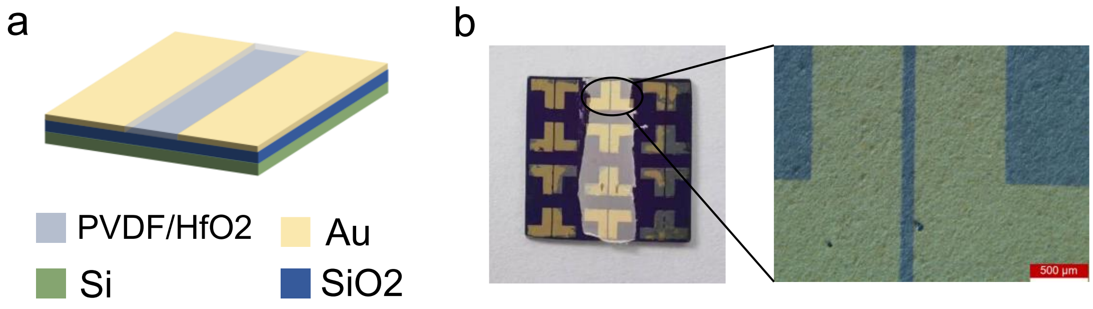

Fig. S10. Device schematics and physical prototype of the self-powered photodetector. (a) Cross-sectional schematic illustrating the layered structure, wherein the electrospun PVDF/HfO_2 nanofiber membrane serves as the light-sensing and piezoelectric active layer. (b) A photograph of the resulting flexible and lightweight device, underscoring its potential for integration into wearable electronics.

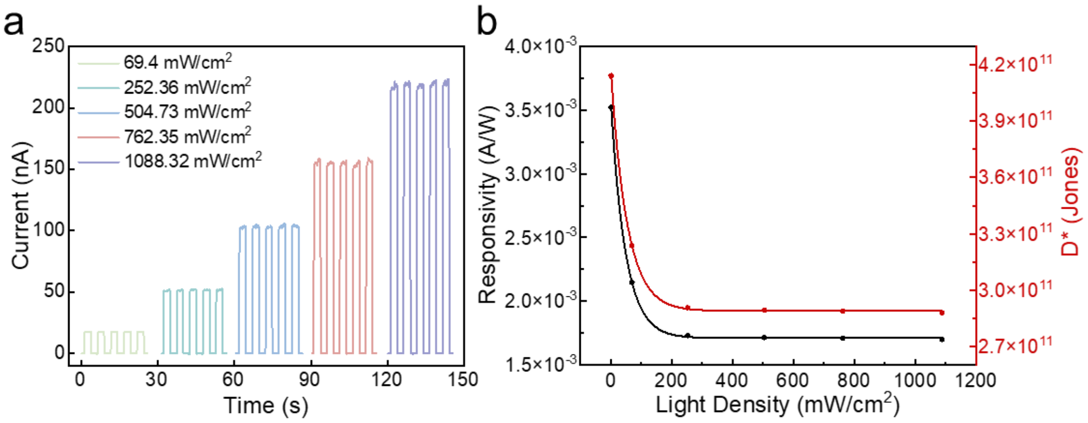

Fig. S11 Performance characterization of the photodetector under 375 nm illumination. (a) Photocurrent response of photodetector (PD) as a function of time under varying light intensities, demonstrating excellent stability and reproducible on/off switching. (b) Corresponding responsivity and specific detectivity (*D**) plotted against incident light intensity, showing the typical decay trend of these key figures of merit with increasing light intensity due to trap-state saturation and enhanced carrier recombination.

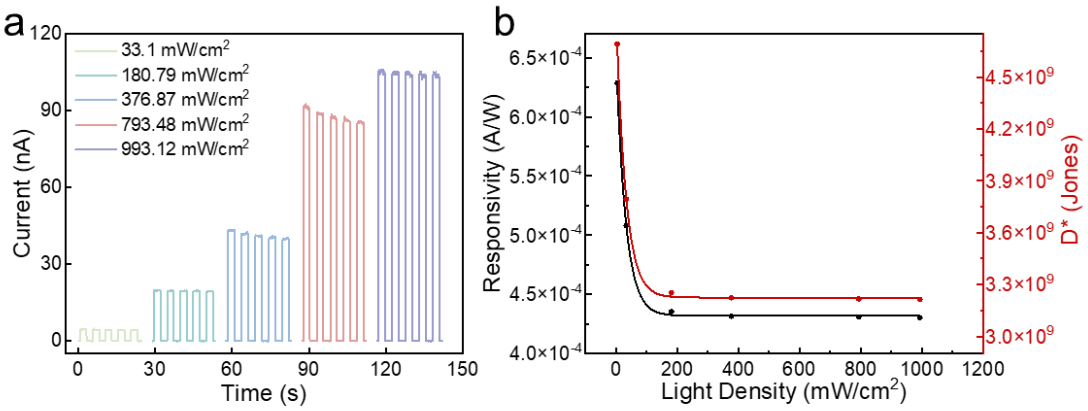

Fig. S12 Photodetection performance under 405 nm illumination. (a) Transient photocurrent response of the photodetector (PD) under varying intensities of 405 nm light, showing stable and reproducible switching cycles. (b) Calculated responsivity and specific detectivity (*D**) as functions of incident light intensity at 405 nm, exhibiting characteristic decay profiles consistent with carrier recombination dynamics at higher photon fluxes.

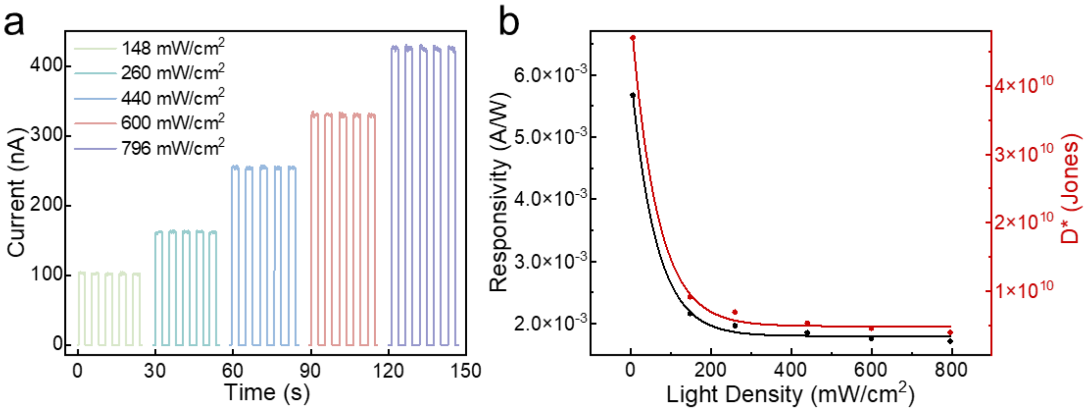

Fig. S13 Wavelength-dependent performance evaluation under 532 nm illumination. (a) Transient photocurrent response of the photodetector (PD) under varying light intensities of 532 nm illumination, demonstrating consistent switching behavior. (b) Derived responsivity and specific detectivity (*D**) as functions of incident light intensity at 532 nm, showing characteristic decay trends that complete the spectral performance analysis of the photodetector.

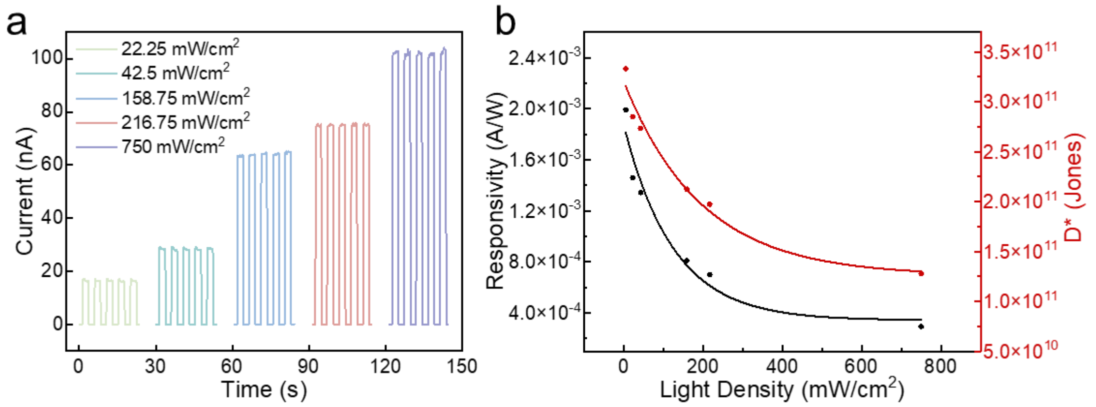

Fig. S14 Extended spectral response characterization under 808 nm near-infrared illumination. (a) Transient photocurrent response under varying light intensities of 808 nm illumination, showing detectable but diminished signals due to approaching the material's bandgap limit. (b) Derived responsivity and specific detectivity (*D**) as functions of light intensity at 808 nm, completing the spectral performance profile and establishing the operational range of the photodetector.

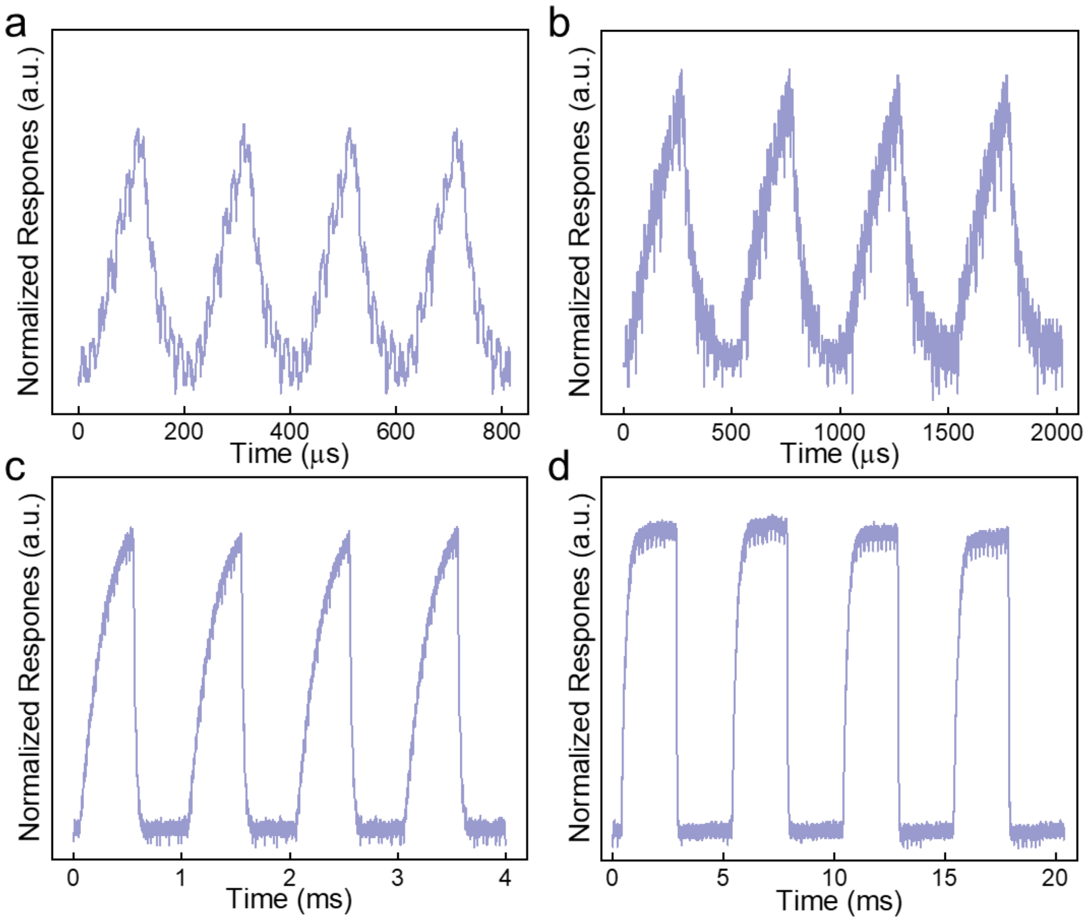

Fig. S15. High-frequency response characterization of the photodetector. Dynamic photocurrent profiles of the photodetector under pulsed illumination with switching intervals of (a) 200 μs, (b) 500 μs, (c) 1 ms, and (d) 5 ms, demonstrating the device's capability for rapid optical switching and establishing its response speed limits.

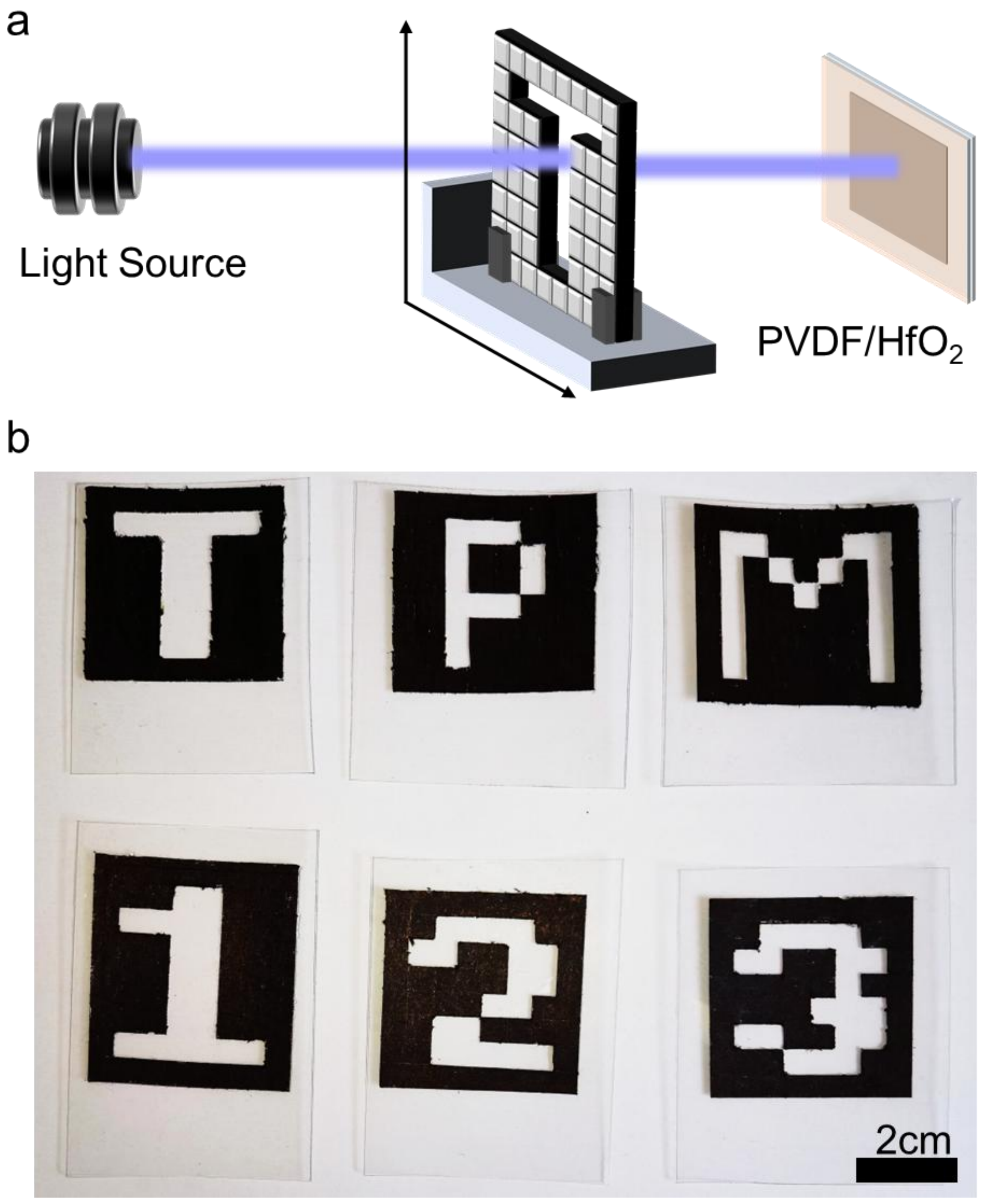

Fig. S16. Experimental setup and substrate characterization. (a) Schematic diagram of custom-built measurement system for piezoelectric/photodetection performance evaluation. (b) Optical microscopy image of the patterned PET substrate with interdigitated electrodes, showing the well-defined electrode architecture used for device fabrication.

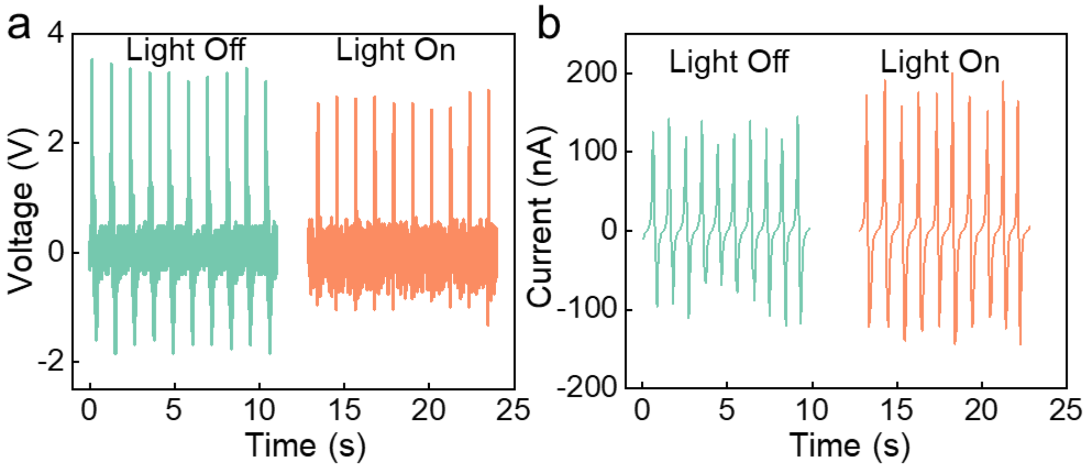

Fig. S17. Optomechanical coupling in piezoelectric response. (a) Output voltage and (b) short-circuit current response of the device under mechanical pressing, measured under illuminated (light-on) and dark (light-off) conditions, demonstrating the modulation of piezoelectric output by photoexcitation.

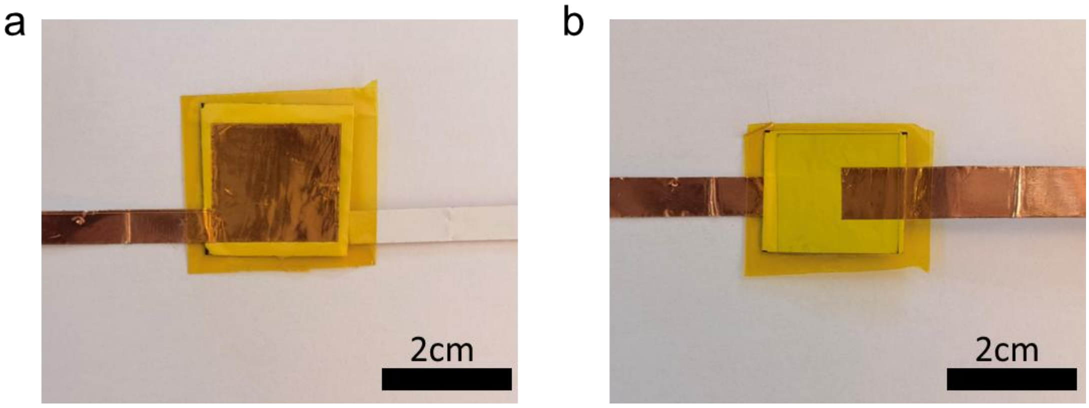

Fig. S18. Physical prototypes demonstrating key attributes for wearable integration. (a) The piezoelectric sensor, illustrating its compact and adaptable form factor. (b) The semi-transparent photodetector based on the nanofiber active layer, underscoring the lightweight and flexible nature of the technology.

Table S1 This work compares the piezoelectric and photoelectric properties with those of other materials.

Sample	Active area (cm^2)	Pressure	Output voltage (V)	Output current (nA)	Responsivity (mA/W)	Specific detection rate (Jones)	References
MXene/PVDF	2.01	200KPa	3.15	134	/	/	[1]
PVDF/PAN	2 × 2	1~20N	3.8	320	/	/	[2]
$BaTiO_3$@P(VDF-TrFE)	2.5 × 2.5	300~700N	6	1520	/	/	[3]
PVDF-TrFE/MXene	1 × 1	18~79N	7	100	/	/	[4]
$BiFeO_3$/PVDF	/	Force through finger knocking	7.6	200	/	/	[5]
PVDF/MWCNT/$BaTiO_3$	2 × 2	1~250KPa	8	/	/	/	[6]
CNF@ZnO/PVDF	2.5 × 1.8	45N	11.8	452	/	/	[7]
MXene/Sm-PMN-PT/PVDF	3 × 3	1~9N	12	1200	/	/	[8]
ZnO@Ag/PVDF	2 × 1	3~22N	15	1775	/	/	[9]
Cs_2SnI_6/PVDF	2 × 2	1~80KPa	9	5000	/	/	[10]
SbSI/PVDF	2.5 × 2.5	2N	5	150	4.5×10^{-3}	/	[11]
$CsPbI_3$/PVDF	3 × 5	10KPa	20	6000	0.3	1.22×10^{9}	[12]
PVDF/CeO_2@PDA	1 × 1	1~100KPa	25.5	350	2.8×10^{-6}	5.21×10^{5}	[13]
**PVDF/HfO_2/nano-copper**	**2 × 2**	**5~30N**	**15**	**355**	**5.67**	**4.14×10^{11}**	**This work**

Data References in Table S1

[1] Zhang J, Yang T, Tian G, et al. Spatially Confined MXene/PVDF Nanofiber Piezoelectric Electronics[J]. Advanced Fiber Materials, 2024, 6(1): 133-144.

[2] Liu Z, Li G, Qin Q, et al. Electrospun PVDF/PAN membrane for pressure sensor and sodium-ion battery separator[J]. Advanced Composites and Hybrid Materials, 2021, 4(4): 1215-1225.

[3] Guan X, Xu B, Gong J. Hierarchically architected polydopamine modified $BaTiO_3$@P(VDF-TrFE) nanocomposite fiber mats for flexible piezoelectric nanogenerators and self-powered sensors[J]. Nano Energy, 2020, 70: 104516.

[4] Wang S, Shao H-Q, Liu Y, et al. Boosting piezoelectric response of PVDF-TrFE via MXene for self-powered linear pressure sensor[J]. Composites Science and Technology, 2021, 202: 108600.

[5] Ichangi A, Lê K, Queraltó A, et al. Electrospun $BiFeO_3$ Nanofibers for Vibrational Energy Harvesting Application[J]. Advanced Engineering Materials, 2022, 24(7): 2101394.

[6] Li J, Yin J, Wee M G V, et al. A Self-Powered Piezoelectric Nanofibrous Membrane as Wearable Tactile Sensor for Human Body Motion Monitoring and Recognition[J]. Advanced Fiber Materials, 2023, 5(4): 1417-1430.

[7] Zhu Q, Song X, Chen X, et al. A high performance nanocellulose-PVDF based piezoelectric nanogenerator based on the highly active CNF@ZnO via electrospinning technology[J]. Nano Energy, 2024, 127: 109741.

[8] Su Y, Li W, Cheng X, et al. High-performance piezoelectric composites via β phase programming[J]. Nature Communications, 2022, 13(1): 4867.

[9] Ma Y, Liu M, Feng Y, et al. ZnO@Ag modified piezoelectric fibers for higher sensitivity and enhanced energy harvesting[J]. Journal of Materials Research and Technology, 2022, 20: 2689-2704.

[10] Mallick Z, Saini D, Sarkar R, et al. Piezo-phototronic effect in highly stable lead-free double perovskite Cs_2SnI_6-PVDF nanocomposite: Possibility for strain modulated optical sensor[J]. Nano Energy, 2022, 100: 107451.

[11] Purusothaman Y, Alluri N R, Chandrasekhar A, et al. Photoactive piezoelectric energy harvester driven by antimony sulfoiodide (SbSI): A $A_VB_{VI}C_{VII}$ class ferroelectric-semiconductor compound[J]. Nano Energy, 2018, 50: 256-265.

[12] Maity K, Pal U, Mishra H K, et al. Piezo-phototronic effect in highly stable $CsPbI_3$-PVDF composite for self-powered nanogenerator and photodetector[J]. Nano Energy, 2022, 92: 106743.

[13] Huang S, Zhao X, Hao C, et al. Multifunctional PVDF/CeO_2@PDA nanofiber textiles with piezoelectric and piezo-phototronic properties for self-powered piezoelectric sensor and photodetector[J]. Chemical Engineering Journal, 2024, 482: 148950.
